# Integrated mechanistic and serological analyses reveal determinants of antibody-dependent enhancement in respiratory coronaviruses

**DOI:** 10.3389/fimmu.2026.1967945

**Published:** 2026-10-23

**Authors:** Tingting Xu, Yu Jiang, Chenjie Li, Huaying Chen, Zhipeng Zhang, Heng Zhang, Yunliang Qiu, Lu Zhou

**Affiliations:** 1 Pharmacological Evaluation Research Center, China State Institute of Pharmaceutical Industry, Shanghai, China; 2 Shanghai InnoStar Bio-tech Co., Ltd. (InnoStar), Shanghai, China

**Keywords:** antibody-dependent enhancement, cytokine, epidemiology, Fc receptor, human serum, MERS-CoV, pseudovirus, SARS-CoV-2

## Abstract

**Background:**

Antibody-dependent enhancement (ADE) is an important concern in viral infection, vaccine development, and antibody-based therapeutics. Although extensively studied in flaviviruses, the molecular mechanisms and epidemiological relevance of ADE in respiratory coronaviruses remain incompletely understood. This study aimed to investigate the epitope-specific mechanisms, cellular pathways, and population characteristics of ADE mediated by SARS-CoV-2 and MERS-CoV antibodies.

**Methods:**

SARS-CoV-2 and MERS-CoV pseudovirus ADE models were established using Fcγ receptor-expressing cells. The effects of epitope-specific antibodies, Fc–FcR interaction blockade, and inhibitors targeting Fyn, neuropilin-1 (NRP1), endosomal acidification, cathepsins, and PIKfyve were evaluated. A CD32B-based ADE model was developed for retrospective analysis of 530 human serum samples collected from 2019 to 2026. Cytokine profiles were analyzed in 175 serum samples. Correlations between antibody levels, ADE and inflammatory markers were statistically evaluated.

**Results:**

Antibodies targeting specific epitopes within coronavirus spike proteins induced ADE of both SARS-CoV-2 and MERS-CoV pseudoviruses in FcγR-expressing cells. ADE was abolished by blocking Fc–FcR interactions, confirming Fc receptor dependence. ADE induction showed a concentration- and viral load-dependent pattern, with higher viral loads requiring increased antibody concentrations for maximal enhancement. Pharmacological inhibition revealed substantial overlap between ADE and classical receptor-mediated viral entry pathways, involving Fyn signaling, NRP1, endosomal acidification, cathepsins, and PIKfyve. Analysis of clinical serum samples demonstrated a significant association between antibody levels and ADE, with ADE incidence peaking during 2023 and remaining elevated thereafter. Serum samples capable of inducing ADE exhibited altered levels of TNF-α and IL-12p70, indicating that there may be enhanced activation of Th1 and increased pro-inflammatory responses.

## Introduction

Frequent cross-species transmission of coronaviruses poses an ongoing threat of high pathogenicity to humans, and the SARS-CoV-2 pandemic has further highlighted the urgency and complexity of prevention and control efforts (1, 2). Although vaccination and antibody-based therapies are central strategies for combating viral epidemics (3), accumulating studies have revealed that virus-specific antibodies may facilitate viral entry into host cells upon binding to the virus and thereby enhance viral infectivity. This antibody-dependent enhancement (ADE) effect has thus become one of the major challenges in vaccine development and clinical translation. Previous efforts to develop vaccines against respiratory syncytial virus (RSV) and dengue virus (DENV) have already resulted in clinical safety incidents and trial failures due to ADE (4, 5).

ADE is mediated by multiple mechanisms, including Fc receptors and the complement system; among these, the Fc-mediated pathway is the most frequently reported and the most common mechanism underlying ADE (6). There are significant differences in the FcγR subtypes involved in ADE among different viruses; for example, SARS-CoV-2 is associated with FcγRIIa and FcγRIIb, while MERS-CoV has been reported to be mediated solely by FcγRIIa (7–9). Studies on SARS-CoV-2 have shown that, in addition to RBD-targeting antibodies, NTD-specific antibodies can also promote viral entry into cells (9). In contrast, ADE in MERS-CoV is mediated solely by neutralizing antibodies targeting the spike RBD, indicating that antibody epitope specificity plays a critical role in ADE (8). The ADE study found that the effects of antibodies targeting the same epitope vary with concentration: moderate concentrations exacerbate infection, while extremely low or high concentrations are relatively safe. This phenomenon does not follow a dose-dependent pattern, as has been confirmed in *in vitro* cell experiments (10).

The molecular mechanisms underlying ADE-mediated entry of the coronavirus are still being investigated, particularly the similarities and differences between this process and receptor-dependent viral entry pathways. Studies have shown that antibody-dependent SARS-CoV infection enters immune cells via an FcγR pathway that is independent of pH and cysteine proteases (11). Interestingly, Yushun Wan et al. (8), in their study of ADE, found that the DPP4-dependent and Mersmab1-dependent MERS-CoV entries can both be activated by proprotein convertases, cell surface proteases, and lysosomal proteases, suggesting that the viral entry mechanisms underlying coronavirus ADE are complex and multifaceted.

Previous clinical reports have shown that ADE can lead to a rapid worsening of patients’ condition following COVID-19 vaccination or monoclonal antibody treatment (12–14). Importantly, antibodies isolated from convalescent patients can induce ADE *in vitro* via the FcγR pathway, and ADE in patients is associated with serum antibody titers and *in vitro* neutralizing activity (15–17). ADE is a complex immune process that can trigger antibody-dependent inflammatory enhancement (ADEI) (18). Previous studies have shown that the occurrence of ADE in severe dengue fever is accompanied by a cytokine storm, in which pro-inflammatory factors such as IL-1β, IL-6, and TNF-α are significantly elevated, and this is closely associated with vascular leakage and disease severity (19, 20). However, research on ADE-associated cytokines in SARS-CoV-2 remains unclear.

This study focuses on ADE of respiratory coronaviruses, systematically investigating FcγR subtypes, antibody epitope specificity, the dose-response relationship between antibodies and viruses, and viral entry pathways. Through the retrospective collection and analysis of human serum samples from 2019 to 2026, the study explored the key influencing factors of ADE, and innovatively detected cytokines in serum samples, ultimately confirming that ADE is a complex immune process within the body.

## Materials and methods

### Cells

Chinese hamster ovary (CHO) cells, as well as CHO cells expressing FcγRIIA (CHO-CD32A), FcγRIIB (CHO-CD32B), and FcγRIIC (CHO-CD32C), were cultured in DMEM/F12 supplemented with 10% heat-inactivated fetal bovine serum (FBS; Gibco), 100 U/ml penicillin and 0.1 mg/ml streptomycin (Gibco) and incubated at 37 °C in 5% CO_2_. THP-1 cells (human macrophages) were cultured in RPMI-1640 medium containing 10% heat-inactivated fetal bovine serum, supplemented with 50 μM β-mercaptoethanol. HEK293T cells, the stably transfected HEK293T-DPP4 (from Yisheng Biotechnology Co., Ltd.) and HEK293T-ACE2&TMPRSS2 (from SinoBiological, Inc.) were cultured in DMEM (Gibco) supplemented with 10% FBS, 100 U/ml penicillin, and 0.1 mg/ml streptomycin (Gibco), and maintained at 37 °C in a 5% CO_2_ atmosphere.

CHO-FcγR cells were constructed by expressing the FcγR genes (CD32A, GenBank accession number: NM-021642; CD32B, GenBank accession number: NM-004001; CD32C, GenBank accession number: NM-201563) were cloned onto the pLV-CMV-MCS-EF1-ZsGreen1-T2A-Puro vector, and the target plasmids were obtained by transforming DH5α competent cells; psPAX2, pMD2.G, and the target plasmid in a 2:1:1 ratio into 293T cells. The supernatant was collected 48 hours post-transfection and used to seed CHO cells. Transduced cells stably expressing FcγR were selected, and the CHO-FcγR cells were maintained in 3 μg/ml puromycin (Sigma-Aldrich).

### Serum samples

A total of 530 serum samples were used, including 28 from 2019 volunteers (14 males and 14 females), 24 from 2020 volunteers (12 men and 12 women), 99 from volunteers in 2021 (53 males, 46 females), 66 from volunteers in 2022 (28 males, 38 females), 100 from volunteers in 2023 (48 males, 52 females), 134 from volunteers in 2024 (70 males, 64 females), 49 from the 2025 volunteer cohort (24 males, 25 females), and 30 from the 2026 volunteer cohort (18 males, 12 females). All volunteers signed informed consent forms. The protocol was approved by the Ethics Committee (see **Table 1**) and complies with all ethical regulations governing human research.

**Table 1:** Summary of ethics committee forms for human serum samples.

Year of serum collection	Approval number	Ethics committee
2019	WFGP-2019-LS PJ-078	Ethics Committee for Clinical Trials at Weifang High-Tech Industrial Development Zone People’s Hospital
2020	WFGP-2020-LS PJ-092	Ethics Committee for Clinical Trials at Weifang High-Tech Industrial Development Zone People’s Hospital
2021	WFGP-2021-LS PJ-075	Ethics Committee for Clinical Trials at Weifang High-Tech Industrial Development Zone People’s Hospital
2022	WFGP-2022-LS PJ-002	Ethics Committee for Clinical Trials at Weifang High-Tech Industrial Development Zone People’s Hospital
2023	K2023-003-01	Ethics Committee for Clinical Trials at Hunan Yiyang Kangya Hospital
2024	FE-GCP-LS-2023-019-T	Ethics Committee for Clinical Trials at Hunan Women and Children’s Hospital
2025	FE-YX-LS-2025-004-T	Ethics Committee for Clinical Trials at Hunan Women and Children’s Hospital
2026	FE-YX-LS-2026-013-T	Ethics Committee for Clinical Trials at Hunan Women and Children’s Hospital

### Analysis of antibody binding to the coronavirus spike protein

Pre-coat the plate overnight at 2–8 °C with respiratory coronavirus S protein (0.25 μg/ml), then block for 3 hours at 37 °C with PBS containing 5% skim milk and 0.05% Tween 20. Dilute the respiratory coronavirus antibody (**Table 2**) to 1 μg/ml, then perform serial 2-fold dilutions in 1% BSA-PBS. Dilute the antibody 1:19 in 1% BSA-PBS, then divide the solution into two aliquots. Detect bound antibodies using horseradish peroxidase (HRP)-labeled goat anti-mouse IgG Fc (HRP) (ABCAM) and HRP-labeled goat anti-rabbit IgG Fc (HRP) (ABCAM). Add 3,3’,5,5’-tetramethylbenzidine (TMB; SOLARBIO) to visualize the reaction, and measure the optical density (OD) at 450 nm (**Table 3**).

**Table 2:** Summary information of antibodies utilized in experiments.

Antibody	Isotype	Immunogen	Source	Identifier
SARS-CoV-2 Spike RBD Antibody	IgG2A	Ala319-Phe541	R&D Systems	Catalog #: MAB105802
SARS-CoV-2 Spike S1 Rabbit pAb	IgG	Val11-Arg682	ABclonal	Catalog #: A20136
Spike S1 Rabbit mAb	IgG	Val200-Gln300	ABclonal	Catalog #: A20834
SARS-CoV-2 Spike Rabbit pAb	IgG	Gln600-Lys700	ABclonal	Catalog #: A20137
SARS-CoV-2 Spike S2 Rabbit pAb	IgG	Gln1174-Pro1273	ABclonal	Catalog #: A20284
SARS-CoV-2 Spike S2 ECD Rabbit pAb	IgG	Ser686-Thr1214	ABclonal	Catalog #: A20138
SARS-CoV-2 Membrane Antibody	IgG2B	Met1-Asn21	R&D Systems	Catalog #: MAB11038
SARS-CoV-2 Membrane Antibody	IgG2B	Arg101-Gln222	R&D Systems	Catalog #: MAB10690
SARS-CoV-2 Envelope Antibody	IgG1	Thr35-Val75	R&D Systems	Catalog #: MAB10925
SARS-CoV-2 Nucleocapsid Antibody	IgG1	Met1-Ala419	R&D Systems	Catalog #: MAB104741
MERS-CoV Spike RBD Antibody	IgG2b	Glu367-Tyr606	R&D Systems	Catalog #: MAB107071
MERS Coronavirus Spike Protein Recombinant Rabbit Monoclonal Antibody (723)	IgG	Met 1-Trp1297	Thermo Fisher	Catalog #: MA5-29975
MERS Coronavirus Spike Protein S2 Monoclonal Antibody	IgG1	Asp726-Pro1296	Thermo Fisher	Catalog #: MA5-29978
MERS-CoV Nucleocapsid Antibody	IgG2b	Met1-Thr411	R&D Systems	Catalog #: MAB10729
MERS Coronavirus M Polyclonal Antibody	IgG	Ser170-Ala219	Thermo Fisher	Catalog #: PA5-112043
MERS Coronavirus Envelope Protein Polyclonal Antibody	IgG	Val6-Try82	Thermo Fisher	Catalog #: PA5-143455
Anti-CD32B Antibody- C-terminal	IgG	\	Abcam	Catalog #ab151497
Anti-CD32A Antibody	IgG	\	Abcam	Catalog #ab175030

**Table 3:** Summary information of proteins utilized in experiments.

Protein	Source	Identifier
SARS-CoV-2 S protein (D614G)	ACRO	Catalog #SPN-C52H3
MERS Spike protein trimer (R748A, R751A, V1060P, L1061P), His Tag (MALS verified)	ACRO	Catalog #SPN-M52H5
Goat Anti-Rabbit IgG Fc (HRP)	ABCAM	Catalog #ab97200
Goat Anti-Mouse IgG Fc (HRP)	ABCAM	Catalog #ab97265
CD32a/Fc gamma RIIA Antibody	SinoBiological, Inc	Catalog #10374-RP02
CD32b/FCGR2B Antibody	SinoBiological, Inc	Catalog #10259-MM02
Peroxidase AffiniPure^®^ Goat Anti-Human IgM, Fc5μ fragment specific	Jackson Immuno Research	Catalog #109-035-043
Peroxidase AffiniPure^®^ Goat Anti-Human IgG (H+L)	Jackson Immuno Research	Catalog #109-035-003
Human IgG	YEASEN	Catalog #36110ES08

### Analysis of human serum antibody binding to the coronavirus spike protein

Pre-coat the plate overnight at 2–8 °C with respiratory coronavirus S protein (0.5 μg/ml), then block for 3 hours at 37 °C using PBS containing 5% skim milk powder and 0.05% Tween 20. Human serum samples were diluted 1:100 in PBS containing 1% skim milk powder and 0.05% Tween 20, then divided into two aliquots. Binding antibodies were detected using horseradish peroxidase (HRP)-labeled goat anti-human immunoglobulin (H+L) (Jackson Immuno Research) and HRP-labeled goat anti-human IgM, Fc5μ (Jackson Immuno Research). Add 3,3’,5’,5’-tetramethylbenzidine (TMB; Sigma-Aldrich) to visualize the reaction, and measure the optical density (OD) at 450 nm. The serum-negative control consisted of a mixture of serum samples from 10 individuals collected in October 2019 and 2020; these samples tested negative in serum-S protein binding assays, neutralization assays, and ADE assays for respiratory coronaviruses.

### Neutralization assay

Antibodies targeting different epitopes of respiratory coronaviruses were serially diluted (5-fold) in 10% FBS/DMEM. Mix the diluted antibodies with SARS-CoV-2 Spike (D614G) pseudotyped virus (titer: 3 × 10^8^ TU/ml, from Jiman Biotechnology Co., Ltd.) or MERS-CoV pseudotyped virus (copy number: 1 × 10¹³ copies/ml, from Yisheng Biotechnology Co., Ltd.) (**Table 4**), incubate for 1 hour, and then inoculate into HEK293T-ACE2&TMPRSS2 or HEK293T-DPP4 cells cultured in 96-well plates. After 48 hours of incubation, measure the chemiluminescence signal (RLU) using a firefly luciferase assay kit (Promega), and calculate the inhibition rate as (1 – RLU value of the test sample/RLU value of the control well (virus only)) × 100%.

**Table 4:** Summary information of viruses utilized in experiments.

Viral	Source	Viral titer	Identifier
SARS-CoV-2 Spike (D614G) Pseudotyped Virus	Genomeditech	3*10^8^TU/ml	Catalog #GM-0220PV14
MERS-CoV Pseudotyped Virus	YEASEN	1E13 Copies/ml	Catalog #18038ES50

Serum samples were diluted 10-fold with 10% FBS/DMEM. The diluted serum was mixed with SARS-CoV-2 Spike (D614G) pseudotyped virus, incubated at 37 °C for 1 hour, and then seeded onto HEK293T-ACE2&TMPRSS2 cells cultured in a 96-well plate. After 48 hours of incubation, RLU was measured using a firefly luciferase assay kit (Promega), and the serum sample inhibition rate was calculated as (1 – RLU value of the test sample/RLU value of the negative serum control well) × 100%.

### Antibody-mediated ADE assays targeting different epitopes

Antibodies targeting different epitopes of respiratory coronaviruses were serially diluted (5-fold) in 10% FBS-DMEM/F12. Mix the diluted antibodies with SARS-CoV-2 Spike (D614G) pseudotyped virus (titer: 3 × 10^8^ TU/ml, from Jiman Biotechnology Co., Ltd.) or MERS-CoV pseudotyped virus (copy number: 1 × 10¹³ copies/ml, from Yisheng Biotechnology Co., Ltd.), incubate for 1 hour, and then inoculate onto CHO-CD32A and CHO-CD32B cells cultured in 96-well plates. After 48 hours of incubation, measure the chemiluminescence signal (RLU) using a firefly luciferase assay kit (Promega). Set the RLU value of the antibody-negative wells to 100% and calculate the relative infection rate.

### Fc receptor-mediated ADE assays

To detect FcγR-mediated ADE in SARS-CoV-2 and MERS-CoV antibodies, pseudotyped virus was mixed with a series of antibody dilutions (5-fold dilutions), incubated for 1 hour, and then seeded onto cells overexpressing FcγR (CHO-CD32A, CHO-CD32B, CHO-CD32C) and THP-1 cells. Luminescence was measured after 48 hours using a firefly luciferase assay kit (Promega). The RLU value of the antibody-negative wells was set to 100%, and the relative infection rate was calculated.

### Effect of different viral titers on ADE

To assess the effect of different viral titers of SARS-CoV-2 and MERS-CoV on ADE, pseudotyped virus at various dilutions was mixed with a series of antibody dilutions (5-fold dilutions), incubated for 1 hour, and then seeded onto CHO-CD32A and CHO-CD32B cells. Luminescence was measured 48 hours later using a firefly luciferase assay kit (Promega). The RLU value of the antibody-negative wells was set to 100%, and the relative infection rate was calculated.

### Fc receptor inhibitors block pseudovirus entry

CHO-CD32A and CHO-CD32B cells were incubated separately at 37 °C for 1 hour with 50 nM anti-CD32A antibody (ABCAM), 50 nM anti-CD32B antibody (C-terminal) (ABCAM), and 50 nM human IgG (Yisheng Biotechnology (Shanghai) Co., Ltd.). The cells were then used in a pseudovirus entry assay.

### Protease inhibitors block pseudovirus entry

Simply put, using 500 μg/ml of MI-1851 (MCE), 200 μmol/ml of EG00229 (MCE), 100 μg/ml chloroquine (SIGMA), 100 μg/ml ammonium chloride (SIGMA), 200 μmol/ml aloxistatin (MCE), and 100 μmol/ml apilimod (MCE) at 37 °C for 1 hour. The cells were then used in pseudovirus infection assays.

### Analysis of FcγR-mediated ADE in serum samples

A 200-fold dilution of SARS-CoV-2 Spike (D614G) pseudotyped virus was mixed with a series of serum dilutions (serum samples were first diluted 10-fold, then further diluted in a 4-fold series), incubated for 1 hour, and seeded onto CHO-CD32B cells. Luminescence was measured 48 hours later using a firefly luciferase assay kit (Promega). Samples exhibiting relative infectivity greater than 200%, which was nearly equal to the mean ± 3 standard deviations of values obtained from 2019 volunteer control sera, were defined as ADE.

### Cytokine assay

Dilute serum samples two-fold using MSD Diluent 2 in a 1:1 volume ratio. Add the diluted samples to wells of an MSD 10-plex cytokine plate pre-coated with capture antibodies and incubate at 25 °C with shaking at 700 rpm for 2 hours. Add a mixture of 10 cytokine detection antibodies to detect bound antibodies and incubate at 25 °C with shaking at 700 rpm for 2 hours. Next, add 150 μl of color development solution to each well to visualize the reaction. Read the plate using a MESO^®^ Sector S 600 chemiluminescence reader and analyze the data using SoftMax Pro software.

### Comparison of ADE effects in different cells under a fluorescence microscope

After configuring the viruses and antibodies according to **Table 5**, mix them in a 1:1 ratio and incubate them in a 37 °C constant temperature incubator for 1 hour. Then, the virus-antibody mixture was added to each corresponding cell at a concentration of 100 μl per well. After 48 hours of incubation, discard the supernatant, wash with 1 ml PBS, add a 1:1 mixture of formaldehyde and acetone, fix at -20 °C for 20 min, discard the fixative, and observe and photograph under a fluorescence microscope.

**Table 5:** Optimal concentration of antigen-antibody complexes from different cells that trigger ADE.

Cell	SARS-CoV-2		MERS-CoV	
	Virus titer	Antibody concentration (μg/ml)	Virus titer	Antibody concentration (μg/ml)
CD32A cells	Pseudovirus diluted 200-fold	1.6	Pseudovirus diluted 20-fold	0.032
CD32B cells	Pseudovirus diluted 200-fold	0.064	Pseudovirus diluted 200-fold	0.064

### Statistics and analysis

In the statistical analysis, SPSS Statistics software (version 21) was used to assess the correlation between variables using the Chi-square test or Fisher’s exact test with the Phi coefficient for dichotomous variables. Multivariate binary logistic regression was utilized to screen independent predictors of ADE. The correlation strength was defined based on the absolute value of correlation coefficient: weak (< 0.3), moderate (0.3–0.7), and strong (≥ 0.7) was defined as a strong correlation. The level of statistical significance was set at a two-sided *P* < 0.05. We used a two-tailed paired t-test or a two-tailed Mann-Whitney U test for two-group comparisons and utilized GraphPad Prism software (version 11) for graphing.

## Result

### The roles of antibodies targeting different epitopes and FcγR receptors in ADE

To systematically evaluate the antigen-binding specificity of antibodies against different coronavirus structural proteins, we first assessed the binding capacity of each antibody to the corresponding Spike (S) protein. ELISA results showed that antibodies targeting the SARS-CoV-2 Spike S1 region (Val11–Arg682, Val200–Gln300, Gln600–Lys700, and the RBD region Ala319–Phe541) and the Spike S2 region (Ser686–Thr1214) were able to bind effectively to the S protein, whereas antibodies targeting the M, N, and E proteins showed no detectable binding to the S protein (**Figure 1A**). Similarly, in MERS-CoV, among three antibodies targeting different regions of the Spike protein, those recognizing the RBD region (Glu367–Tyr606) and the full-length Spike protein (Met1–Trp1297) exhibited strong binding affinity for the S protein, whereas the antibody recognizing the S2 region (Asp726–Pro1296) showed relatively weak binding affinity; in contrast, antibodies targeting the M, N, and E proteins showed no S-protein binding ability (**Figure 1B**).

**Figure 1:**
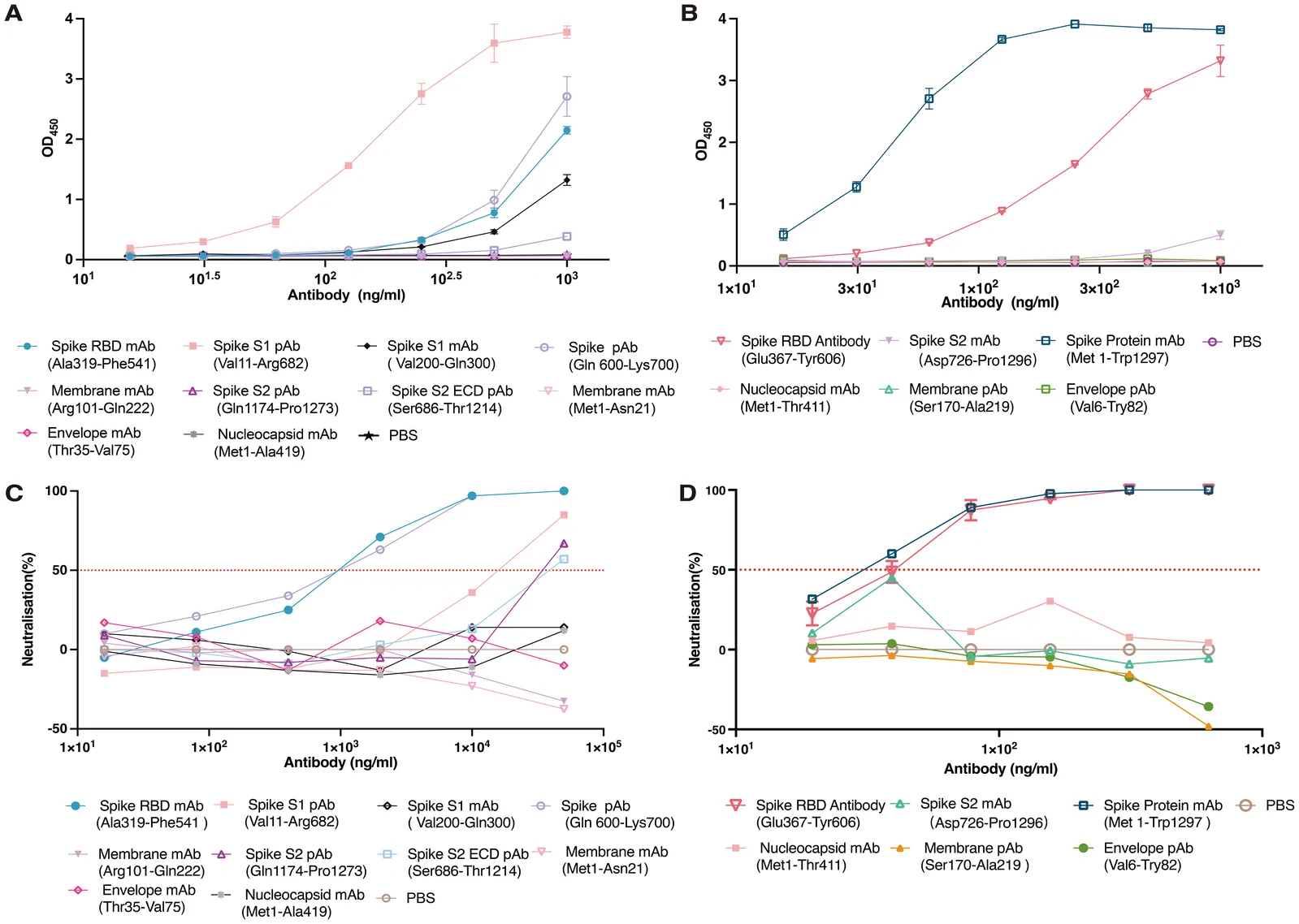
Relationship between the binding of antibodies targeting different epitopes to the respiratory coronavirus spike protein and their neutralizing capacity. **(A)** Binding of SARS-CoV-2 antibodies targeting different epitopes to the SARS-CoV-2 S protein, as determined by ELISA, expressed as mean optical density at 450 nm (OD_450_). **(B)** Binding of MERS-CoV antibodies targeting different epitopes to the MERS-CoV S protein, as determined by ELISA and expressed as mean optical density at 450 nm (OD_450_). **(C)** Neutralization values of SARS-CoV-2 antibodies targeting different epitopes against pseudotyped SARS-CoV-2 D614G variants, with the 50% neutralization values for each antibody indicated. **(D)** 50% neutralization values of MERS-CoV antibodies targeting different epitopes against pseudotyped MERS-CoV variants. **(A, B)**: Dots and error bars indicate the means and standard deviations of double wells, respectively. **(C, D)** Dots and error bars indicate the means and standard deviations of triplicate wells, respectively. Data represent the mean ± SD. Source data are provided as a source data file.

After confirming that different antibodies possess corresponding S-protein-binding capabilities, we further evaluated their ability to inhibit viral infection. Given that SARS-CoV-2 and MERS-CoV rely on ACE2 and DPP4 receptors, respectively, to enter host cells, we used HEK293T cells that overexpress either ACE2&TMPRSS2 or DPP4 and added SARS-CoV-2 Spike (D614G) pseudovirus or MERS-CoV pseudovirus to conduct neutralization assays. (**Figures 1C, D**). The results showed that SARS-CoV-2 antibodies targeting the Spike S1 region (Val11–Arg682, Gln600–Lys700, and the RBD region Ala319–Phe541) and the S2 region (Ser686–Thr1214) of SARS-CoV-2 all exhibited significant neutralizing activity, with neutralizing capacity increasing with rising antibody concentration; in contrast, antibodies targeting the M, N, and E proteins did not demonstrate the ability to inhibit viral infection (**Figure 1C**). For MERS-CoV, antibodies targeting the Spike RBD region (Glu367–Tyr606) and the full-length Spike protein (Met1–Trp1297) exhibited strong neutralizing activity, whereas antibodies targeting the Spike S2 region (Asp726–Pro1296) and the M, N, and E proteins did not show significant inhibition of viral infection (**Figure 1D**). Overall, the results of the pseudovirus neutralization assays were generally consistent with those of the ELISA assays.

To further evaluate the ADE effects mediated by antibodies targeting different epitopes, we established an ADE assay model using CHO cells overexpressing FcγRIIA (CHO-CD32A) and FcγRIIB (CHO-CD32B), respectively (**Figures 2A–D**, **Table 6**). The results showed that, in the SARS-CoV-2 assay system, the Spike RBD monoclonal antibody induced significant ADE effects in both CHO-CD32A and CHO-CD32B cells. In contrast, the Spike S1 polyclonal antibody (Asn11–Arg682) exhibited only weak ADE, while no significant ADE effects were observed for other antibodies targeting different epitopes (**Figures 2A, B**).

**Figure 2:**
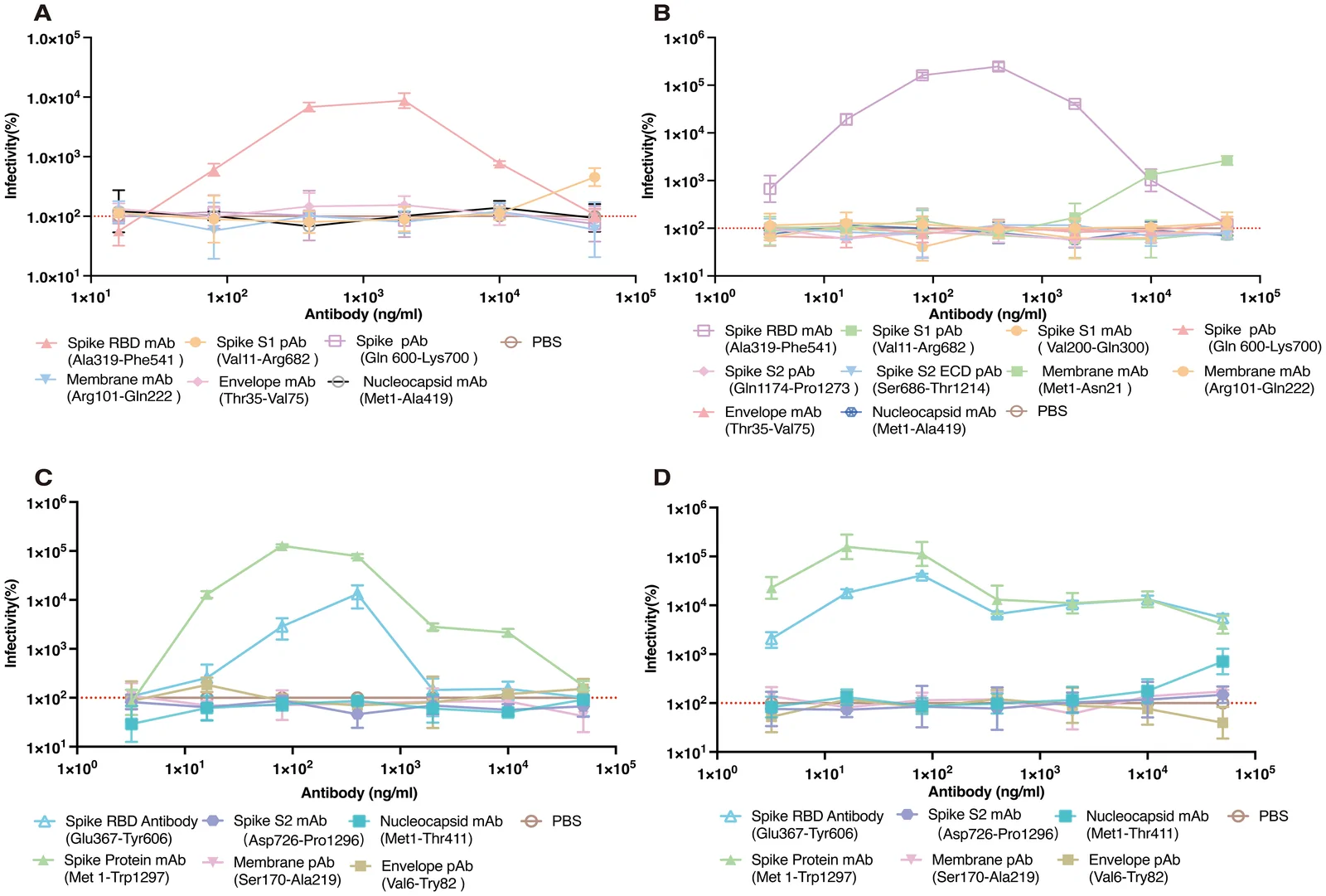
Antibody-mediated enhancement of infection targeting different epitopes. **(A, B)** The effects of SARS-CoV-2 antibodies targeting different epitopes on pseudovirus SARS-CoV-2 D614G infection were assessed in CHO-CD32A **(A)** and CHO-CD32B **(B)**. **(C, D)** The effects of MERS-CoV antibodies targeting different epitopes on pseudovirus MERS-CoV infection were assessed in CHO-CD32A cell **(C)** and CHO-CD32B cell **(D)**. Dots and error bars indicate the means and standard deviations of triplicate wells, respectively. Data represent the mean ± SD. Source data are provided as a source data file.

**Table 6:** Binding, neutralization, and ADE of antibodies targeting distinct epitopes.

Antibody	Epitope	ELISA binding	Neutralization	ADE-CD32A	ADE-CD32B
SARS-CoV-2					
SARS-CoV-2 Spike RBD Antibody	Ala319-Phe541	+	+	+	+
SARS-CoV-2 Spike S1 Rabbit pAb	Val11-Arg682	+	+	+	+
Spike S1 Rabbit mAb	Val200-Gln300	+	-	/	-
SARS-CoV-2 Spike Rabbit pAb	Gln600-Lys700	+	+	-	-
SARS-CoV-2 Spike S2 Rabbit pAb	Gln1174-Pro1273	-	+	/	-
SARS-CoV-2 Spike S2 ECD Rabbit pAb	Ser686-Thr1214	+	+	/	-
SARS-CoV-2 Membrane Antibody	Met1-Asn21	-	-	/	-
SARS-CoV-2 Membrane Antibody	Arg101-Gln222	-	-	-	-
SARS-CoV-2 Envelope Antibody	Thr35-Val75	-	-	-	-
SARS-CoV-2 Nucleocapsid Antibody	Met1-Ala419	-	-	-	-
MERS-CoV					
MERS-CoV Spike RBD Antibody	Glu367-Tyr606	+	+	+	+
MERS-CoV Spike Protein Recombinant Rabbit Monoclonal Antibody (723)	Met 1-Trp1297	+	+	+	+
MERS-CoV Spike Protein S2 Monoclonal Antibody	Asp726-Pro1296	+	-	-	-
MERS-CoV Nucleocapsid Antibody	Met1-Thr411	-	-	-	-
MERS-CoV M Polyclonal Antibody	Ser170-Ala219	-	-	-	-
MERS-CoV Envelope Protein Polyclonal Antibody	Val6-Try82	-	-	-	-

ELISA binding: +: OD_450_ > 0.2, -: OD_450_ < 0.2; Neutralization: +: > 50%, - < 50%; \ADE: +: Infectivity > 200%, -: Infectivity < 200%./: Not tested.

In ADE assays for MERS-CoV, the MERS-CoV Spike RBD mAb (Glu367-Tyr606) and the MERS-CoV Spike Protein mAb (Met1-Trp1297) induced strong ADE in both CHO-CD32A and CHO-CD32B cells (**Figures 2C, D**). In summary, this study demonstrates that antibodies targeting epitopes in the viral RBD region are more likely to induce ADE, and that cells expressing CD32B are more suitable than those expressing CD32A for evaluating ADE in antibodies targeting different epitopes.

Based on the above results, there are significant differences in the ability of antibodies targeting different epitopes to mediate ADE, with RBD-targeting antibodies exhibiting strong ADE. Given that ADE primarily occurs in cells expressing FcγR, we further investigated the role of Fc receptors in this process. To this end, in cell models expressing CD32A and CD32B, respectively, we blocked Fc receptors using anti-CD32A or anti-CD32B-specific antibodies, or competitively bound Fc receptors by exogenously adding IgG, to assess their effects on SARS-CoV-2- and MERS-CoV-mediated ADE. The results showed that both Fc receptor blockade and competitive IgG binding significantly inhibited SARS-CoV-2- and MERS-CoV-induced ADE (**Figures 3A, B**). The above results indicate that the process of ADE in respiratory coronaviruses is highly dependent on the interaction between virus-specific antibodies and Fc receptors; blocking this binding can effectively inhibit the occurrence of ADE.

**Figure 3:**
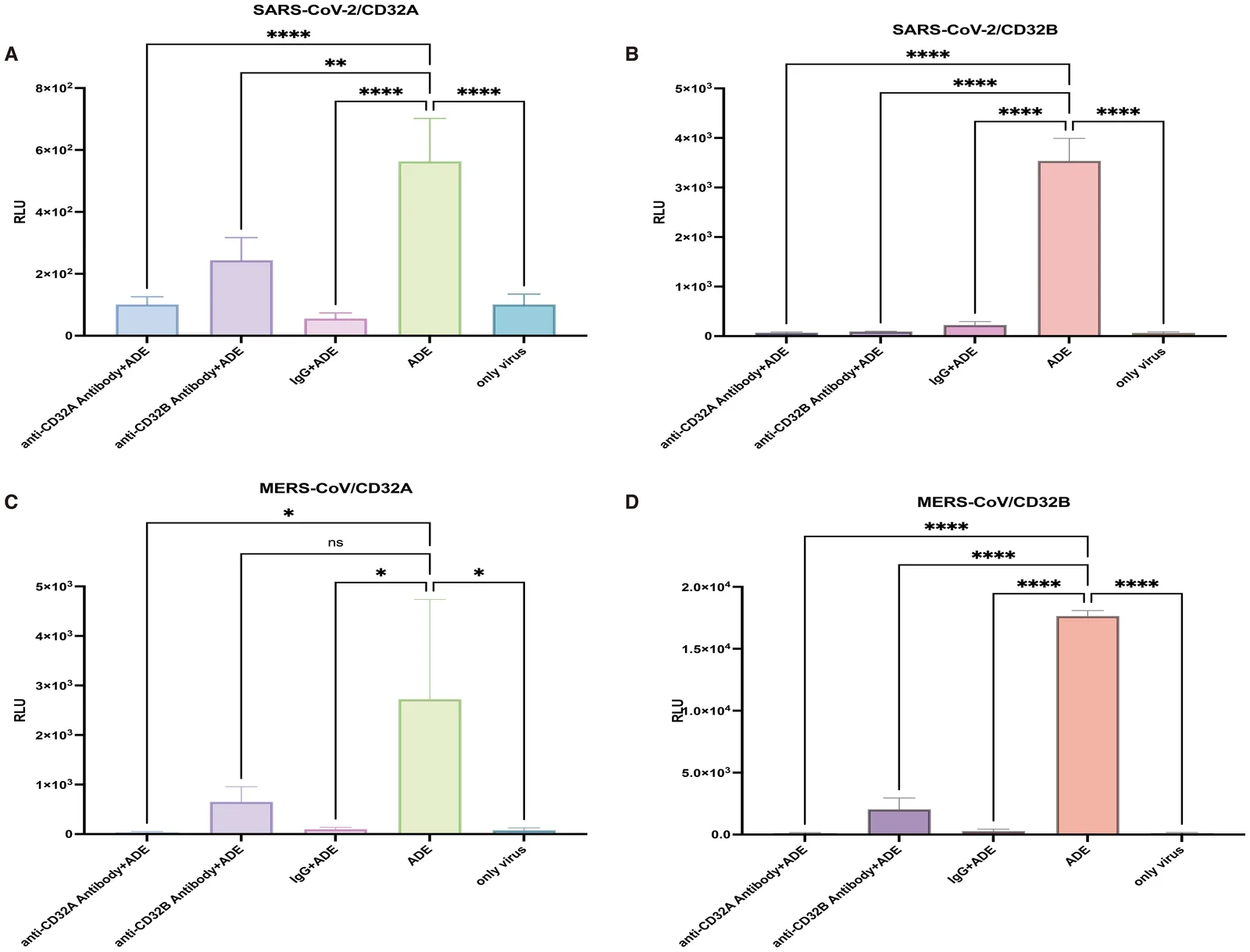
Effects of Fc receptor inhibitors and IgG on ADE. **(A)** Effects of anti-CD32A antibodies, anti-CD32B antibodies and IgG on CD32A-mediated ADE of SARS-CoV-2 and MERS-CoV**; (B)** Effects of anti-CD32A antibodies, anti-CD32B antibodies and IgG on CD32A-mediated ADE of MERS-CoV; **(C)** Effects of anti-CD32A antibodies, anti-CD32B antibodies and IgG on CD32B-mediated ADE of SARS-CoV-2; **(D)** Effects of anti-CD32A antibodies, anti-CD32B antibodies and IgG on CD32B-mediated ADE of S MERS-CoV. Significance is indicated as *P < 0.05; **P < 0.01, ***P < 0.001 and ****P < 0.0001 in one-way ANOVA. Dots and error bars indicate the means and standard deviations of triplicate wells, respectively. Data represent the mean ± SD. Source data are provided in a source data file.

We further investigated the effects of different Fcγ receptor subtypes on the occurrence of ADE. To this end, we established CHO cell models expressing the FcγRII subtypes CD32A, CD32B, and CD32C (**Figures 4A, B**) and validated the results using THP-1 cells, which endogenously express Fc receptors. The results showed that, in the absence of neutralizing antibodies, neither SARS-CoV-2 nor MERS-CoV pseudoviruses could effectively infect FcγR-expressing cells; however, upon addition of RBD-specific neutralizing antibodies, both SARS-CoV-2 and MERS-CoV pseudoviruses significantly infected CHO cells expressing CD32A and CD32B, with the ADE effect mediated by CD32B being the most pronounced (**Figures 5A, C**). In contrast, no obvious ADE was observed in CHO cells expressing CD32C or in THP-1 cells. Furthermore, SARS-CoV-2 and MERS-CoV neutralizing antibodies each promoted infection only by the corresponding pseudovirus and did not promote infection by the heterologous virus, indicating that ADE exhibits strict antigen specificity (**Figures 5B, D**). Fluorescence microscopy further confirmed that virus-specific neutralizing antibodies significantly enhanced the infection of CHO-CD32A and CHO-CD32B cells by SARS-CoV-2 and MERS-CoV pseudoviruses. In contrast, robust pseudovirus infection was observed in receptor-expressing HEK293T-ACE2/TMPRSS2 and HEK293T-DPP4 cells in the absence of antibodies. These findings provide visual evidence that virus-specific antibodies can promote SARS-CoV-2 and MERS-CoV pseudovirus infection in FcγR-expressing cells (**Figure 6**). In summary, in our overexpression cell-based system, SARS-CoV-2- and MERS-CoV-RBD-specific neutralizing antibodies can mediate ADE through Fcγ receptor-dependent pathways, with CD32B contributing substantially to the observed enhancement.

**Figure 4:**
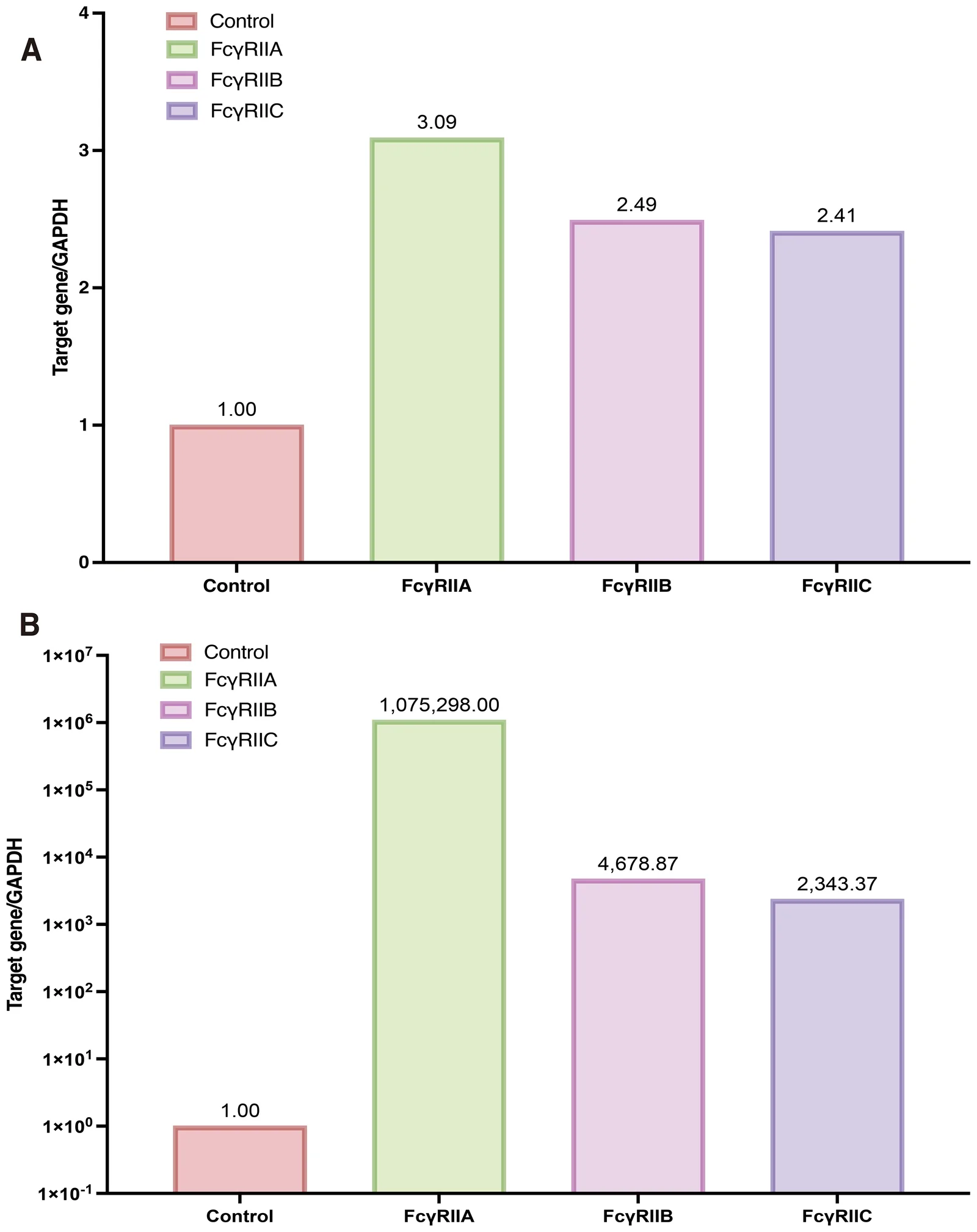
Fc receptor expression and target gene regulation in FcR-overexpressing CHO stable cell lines. **(A)** Fc receptor expression levels in CHO cells. **(B)** Target gene expression levels in different FcR-overexpressing stable cell lines.

**Figure 5:**
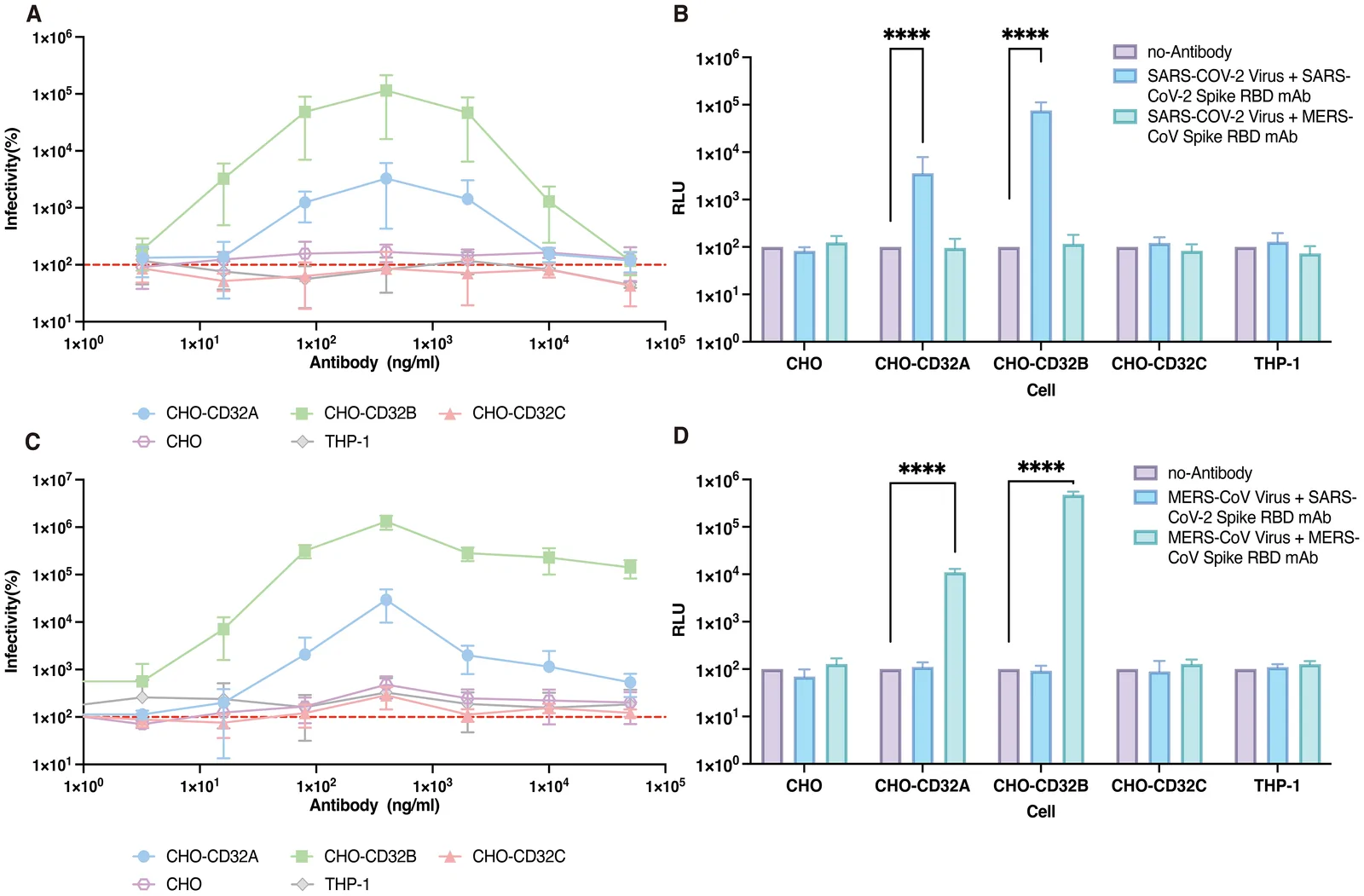
Comparison of ADE intensity mediated by cells expressing different Fc receptors. **(A)** ADE intensity induced by SARS-CoV-2 RBD antibodies in cells expressing different Fc receptors. CHO cells not expressing Fc receptors were used as a control. **(B)** SARS-CoV-2 pseudovirus entry into CHO cells expressing different FcRs, mediated by SARS-CoV-2 Spike RBD mAb and MERS-CoV Spike RBD mAb, with no-antibody as the negative control, ****P < 0.0001 in two-tailed paired t-test. **(C)** ADE intensity induced by MERS-CoV RBD antibodies in cells expressing different Fc receptors. CHO cells not expressing Fc receptors were used as a control. **(D)** SARS-CoV-2 Spike RBD mAb and MERS-CoV Spike RBD mAb-mediated entry of MERS-CoV pseudoviruses into CHO cells expressing different FcRs, ****P < 0.0001 in two-tailed paired t-test. Dots and error bars indicate the means and standard deviations of triplicate wells, respectively. Data represent the mean ± SD. No-antibody was used as the negative control. Source data are provided as a source data file.

Furthermore, to investigate the effects of viral load and antibody concentration on ADE, we assessed ADE in CHO-CD32A and CHO-CD32B cells using various combinations of SARS-CoV-2 and MERS-CoV pseudoviruses at different concentrations and RBD-specific neutralizing antibodies (**Figure 7**). The results showed that as viral load increased, both SARS-CoV-2- and MERS-CoV-mediated ADE effects gradually intensified, suggesting that ADE intensity is positively correlated with viral load. At the same time, the optimal antibody concentration for inducing ADE varied with viral load; as viral load increased, the antibody concentration required to induce the strongest ADE effect also increased accordingly. Furthermore, for both SARS-CoV-2 and MERS-CoV, ADE mediated by CD32B exhibited a broader range of effective antibody concentrations compared to CD32A, suggesting that CD32B has a stronger capacity to mediate ADE under varying antibody concentration conditions.

**Figure 6:**
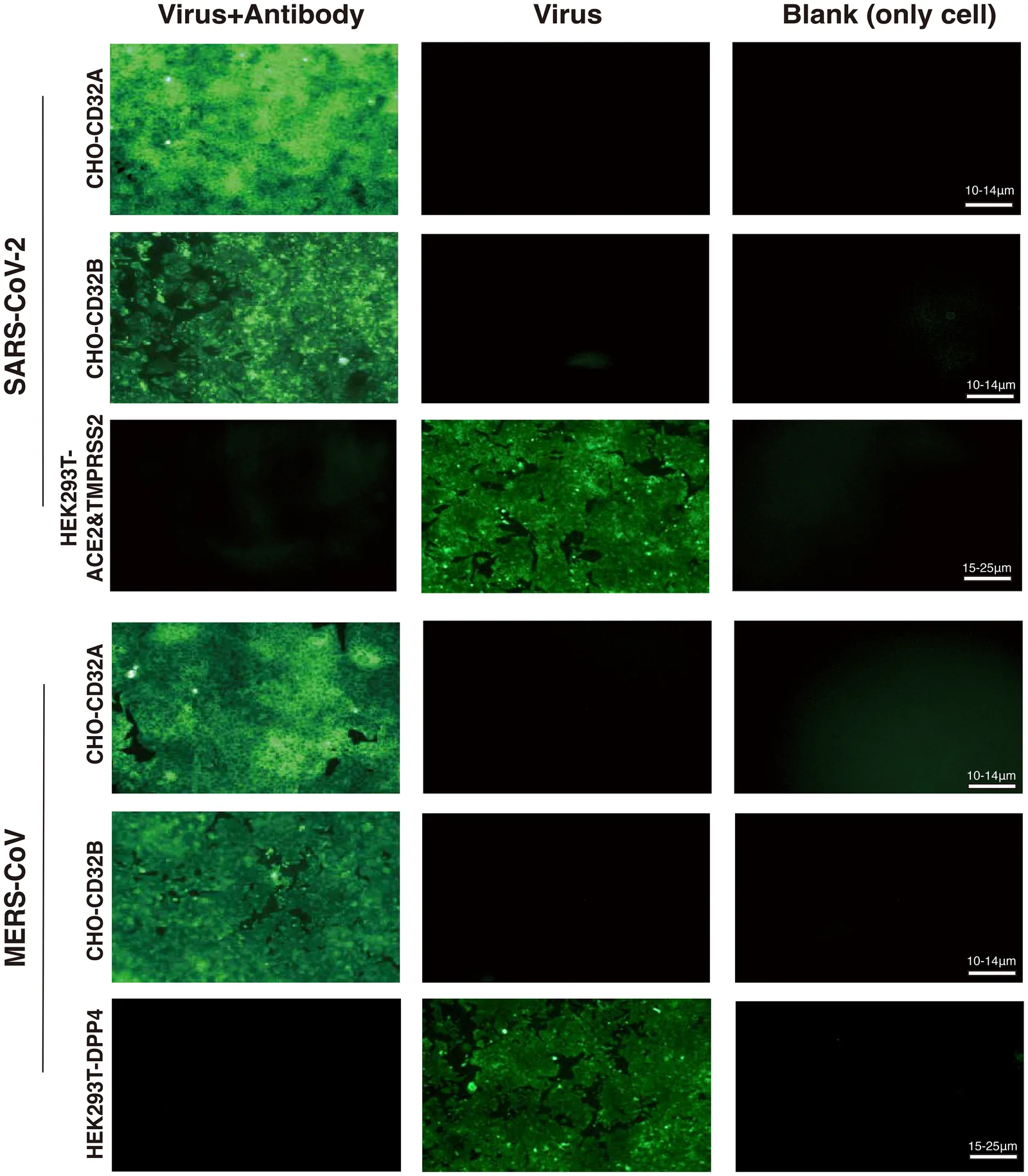
Fluorescence microscopy demonstrates antibody-dependent enhancement of viral infection in Fc receptor-expressing cells.

**Figure 7:**
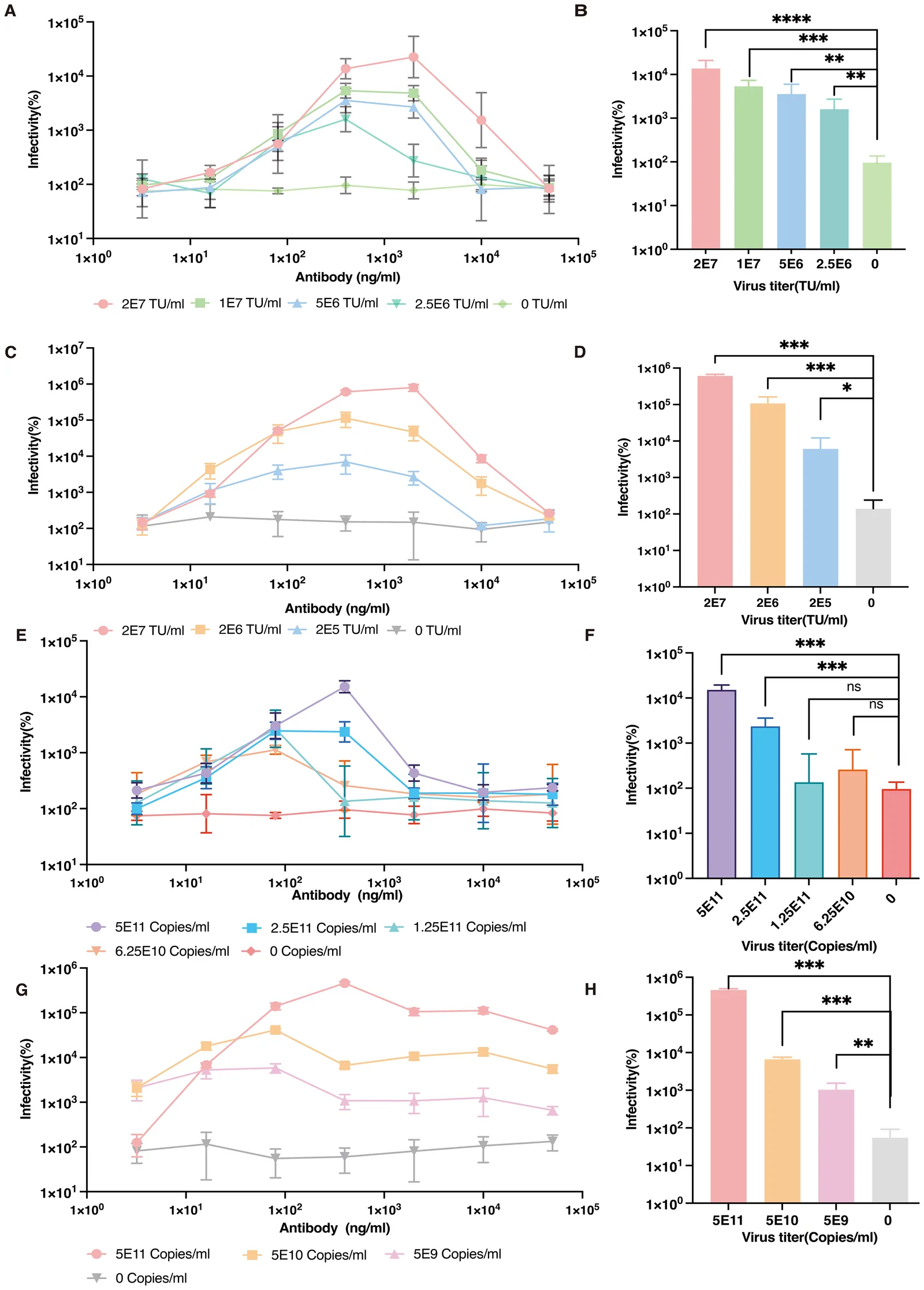
Effects of different viral concentrations on ADE. **(A)** Effects of different SARS-CoV-2 viral titers on CD32A-mediated ADE. **(B)** Effects of different SARS-CoV-2 viral titers on CD32A-mediated ADE in the presence of 400 ng/ml antibody; ***P < 0.001, **P < 0.01 in one-way ANOVA. **(C)** Effects of different SARS-CoV-2 viral titers on CD32B-mediated ADE. **(D)** Effect of different SARS-CoV-2 viral titers on CD32B-mediated ADE in the presence of 400 ng/ml antibody; ***P < 0.001, **P < 0.01, *P < 0.05 in one-way ANOVA. **(E)** Effect of different MERS-CoV viral titers on CD32A-mediated ADE. **(F)** Effect of different MERS-CoV viral titers on CD32A-mediated ADE in the presence of 400 ng/ml antibody; ***P < 0.001 in one-way ANOVA. **(G)** Effect of different MERS-CoV viral titers on CD32B-mediated ADE. **(H)** Effect of different MERS-CoV viral titers on CD32B-mediated ADE in the presence of 400 ng/ml antibody; ***P < 0.001, **P < 0.01 in one-way ANOVA. Dots and error bars indicate the means and standard deviations of triplicate wells, respectively. Data represent the mean ± SD. Source data are provided as a source data file.

### Respiratory coronavirus ADE and the classical route of viral infection

Since RBD-specific antibodies can mediate significant ADE effects via FcγR, we further investigated the molecular mechanisms by which respiratory coronaviruses enter cells via FcγR and conducted analyses using protease inhibitors targeting different stages of viral infection (**Figures 8**, **9**). The results showed that, in the presence of RBD-specific antibodies, the furin inhibitor MI-1851 and the neuropilin-1 (NRP1) inhibitor EG00229 both significantly inhibited the ADE effects and viral infection of SARS-CoV-2 and MERS-CoV pseudoviruses in CHO-CD32A and CHO-CD32B cells (**Figures 8A, B**), suggesting that host proteases and co-receptors are involved in FcγR-mediated viral entry. Meanwhile, ammonium chloride and chloroquine not only inhibited classic receptor-mediated viral entry but also significantly suppressed ADE, indicating that endosomal acidification is a critical step in FcγR-dependent infection (**Figures 8C, D**). Furthermore, the cathepsin inhibitor aloxistatin and the phosphoinositide 3-kinase (PIKfyve) inhibitor apilimod not only inhibited virus entry via classical receptors in the absence of antibodies but also significantly reduced viral infection of CHO-CD32A and CHO-CD32B cells in the presence of RBD-specific antibodies (**Figures 8E, F**), suggesting that cathepsins and PIKfyve are simultaneously involved in both classically receptor-dependent and FcγR-dependent viral entry processes. Overall, although the classically receptor-mediated infection pathway and FcγR-mediated ADE differ in their initiation, they share similar molecular mechanisms in key steps such as intracellular transport, endosomal maturation, and membrane fusion.

**Figure 8:**
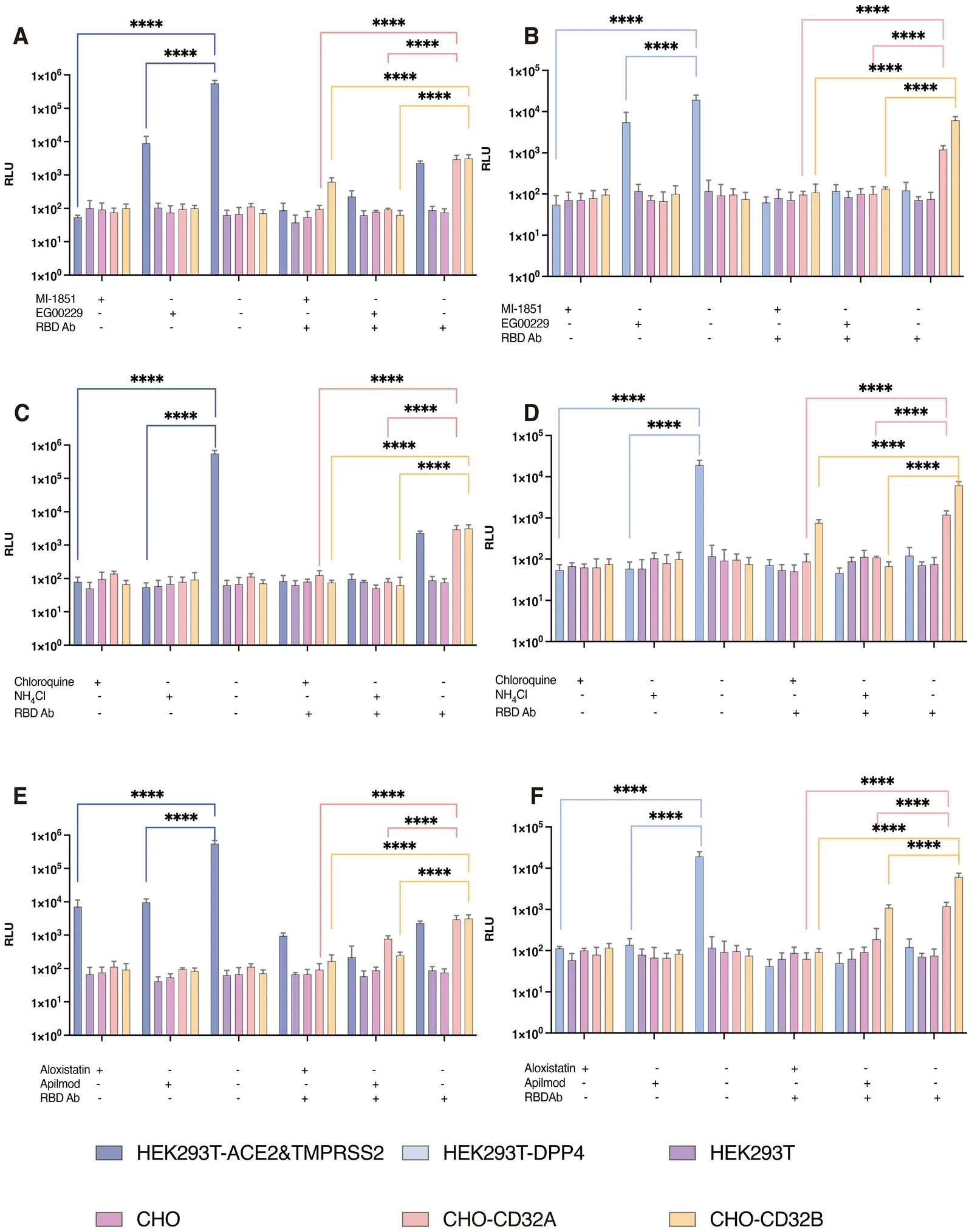
Comparison of antibody-dependent enhancement pathways for coronavirus entry with classical viral infection pathways. (**A, B)** Effects of the furin inhibitor MI-1851 and the NRP1 inhibitor EG00229 on SARS-CoV-2 **(A)** and MERS-CoV **(B)** infection of viral host cells and Fc receptor cells. **(C, D)** Effects of ammonium chloride and chloroquine, which inhibit endosomal acidification, on SARS-CoV-2 **(C)** and MERS-CoV **(D)** infection of target cells and Fc receptor cells. **(E, F)** Effects of the cathepsin inhibitor aloxistatin and the phosphoinositide PIKfyve inhibitor apilimod on SARS-CoV-2 **(E)** and MERS-CoV **(F)** infection of target cells and Fc receptor cells. Significance is indicated by ****P < 0.0001 using one-way ANOVA. Dots and error bars indicate the means and standard deviations of triplicate wells, respectively. Data represent the mean ± SD. Source data are provided in a source data file.

**Figure 9:**
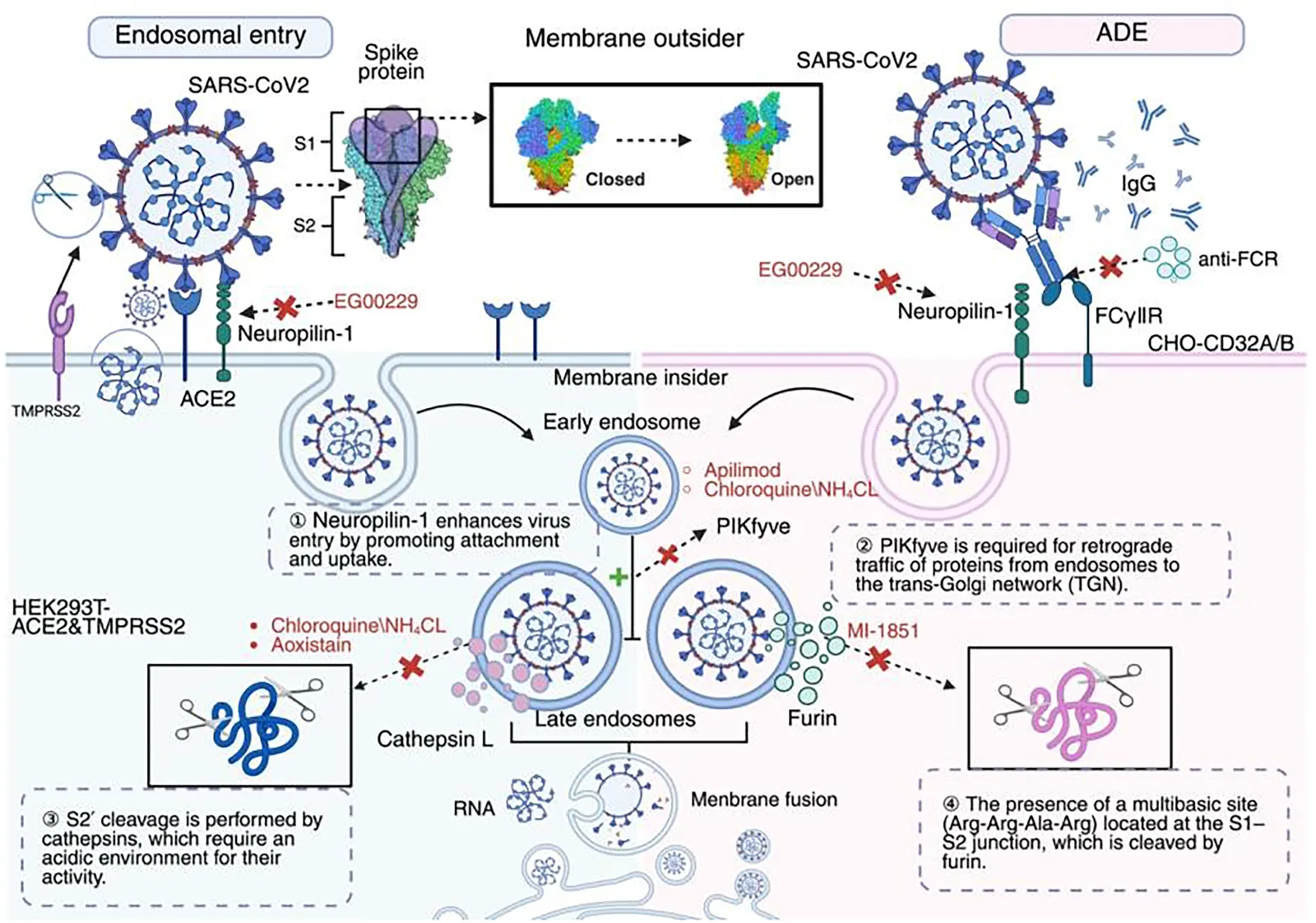
Virus-receptor-mediated and FcγRII-mediated entry pathways of SARS-CoV-2 and endosomal processing. The left (blue) panel illustrates the canonical ACE2-mediated entry pathway of SARS-CoV-2. The viral spike (S) protein binds to the ACE2&TMPRSS2 receptor on the host cell surface, inducing a conformational transition of the receptor-associated complex from a flattened to an upright state, thereby exposing the S2 fusion machinery. NRP1 acts as a host factor that enhances viral attachment and entry efficiency. This process triggers endocytosis, resulting in the formation of early endosomes. Subsequent endosomal maturation involves PIKfyv -dependent trafficking, endosomal acidification, and cleavage by cathepsin L, leading to the formation of late endosomes and the release of viral RNA into the cytoplasm. In the presence of TMPRSS2, S2′ cleavage occurs at the cell surface. Fusion between viral and cellular membranes forms a fusion pore through which viral RNA is released into the host cell cytoplasm for uncoating and replication. The right (pink) panel depicts the FcγRII-mediated antibody-dependent entry pathway. SARS-CoV-2 is first bound by specific IgG antibodies, forming immune complexes. The Fc region of IgG is then recognized by FcγRII on the host cell surface, initiating receptor-mediated endocytosis. NRP1 also contributes to enhanced viral uptake in this pathway. Following internalization, the virus traffics through early endosomes and undergoes PIKfyve-dependent maturation, endosomal acidification, and cathepsin L-mediated processing, resulting in late endosome formation and viral RNA release. Putative targets for inhibition of ADE are indicated in the figure.

### ADE analysis in human serum

To investigate the incidence of ADE across different years and the association between ADE and serum antibodies, we retrospectively collected serum samples from 2019 to 2026 and conducted a systematic study. Neutralizing antibodies were detected using HEK293T-ACE2&TMPRSS2 cells (**Figure 10A**). The results showed that samples from 2019–2020 contained no neutralizing antibodies, while the seropositivity rate for 2021–2025 increased annually with the spread of the virus and remained at a high level, consistent with epidemiological trends, confirming that antibody production is associated with viral exposure (**Figure 10**, **Table 7**). ELISA validation further confirmed the presence of Anti-S-IgG/Anti-S-IgM antibodies against the Spike protein, and the results were largely consistent with the trends observed in the neutralization assays. (**Figures 10B**, **C**). Subsequently, using a CD32B-mediated ADE model, we found that sera from 2019–2020 did not induce ADE at any dilution; in contrast, some sera from 2021–2026 exhibited ADE positivity at specific dilution gradients, confirming that ADE occurrence is antibody concentration-dependent (**Figure 10D**). Notably, although neutralizing activity was not detected in a small number of individuals, Anti-S-IgG was detected by ELISA (**Figures 11A, B**), suggesting that ADE may be mediated by non-neutralizing antibodies or low-titer antibodies. Furthermore, replicate experiments on 55 serum samples from 2024 demonstrated good reproducibility (**Supplementary Figure S1**).

**Figure 10:**
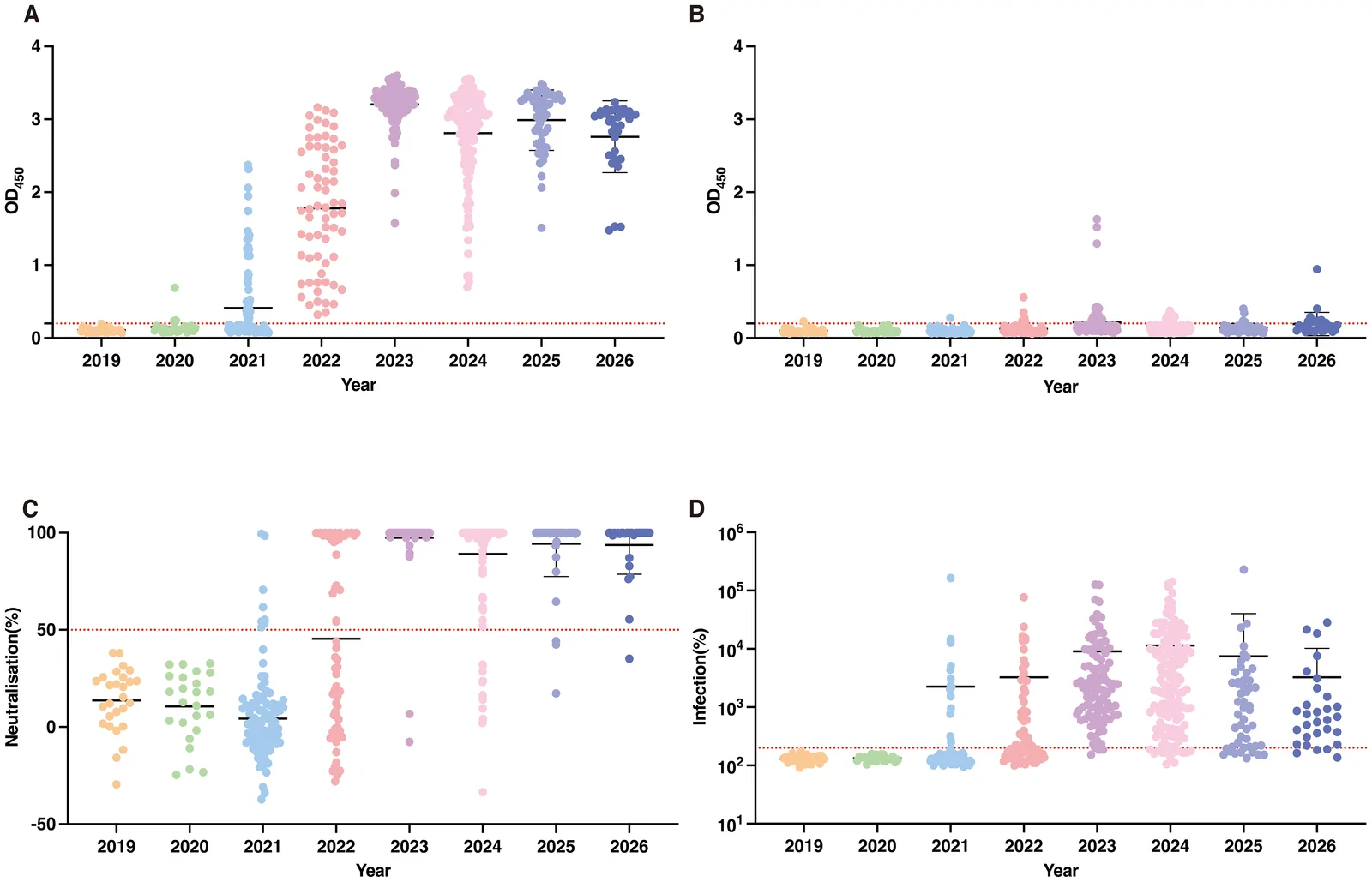
Detection of SARS-CoV-2-specific IgG, IgM, and neutralizing antibodies in human serum samples from 2019 to 2026, and the incidence of ADE caused by these antibodies. **(A, B)** SARS-CoV-2-specific Anti-S-IgG **(A)** and Anti-S-IgM **(B)** antibody testing in human serum: 2019 (n=28), 2020 (n=24), 2021 (n=99), 2022 (n=66), 2023 (n=100), 2024 (n=134), 2025 (n=49), 2026 (n=30); results with OD_450_ > 0.2 were considered positive; **(C)** SARS-CoV-2-specific neutralizing antibody testing in human serum from 2019 to 2026. The cut point 50% was nearly equal to the values of (Mean (inhibition rate, %) + 3SD (inhibition rate, %)), by 2019 volunteer control sera. **(D)** Assessment of the ability of human serum from 2019–2026 to induce ADE. The RLU value of the serum-negative well was set to 100%, and the relative infectivity rate was calculated. A that showed more than 200%, which was nearly equal to the values of average + 3 standard deviations given by 2019 volunteer control sera, was defined as ADE. **(A, B)** Dots and error bars indicate the means and standard deviations of double wells. **(C, D)** Dots and error bars indicate the means and standard deviations of triplicate wells. Data represent the mean ± SD. Source data are provided as a source data file.

**Table 7:** SARS-CoV-2 antibody and ADE assay in human serum (2019–2026).

Year	ADE	Neutrali-zation	Anti-S-IgG	Anti-S-IgM	ADE & Neut-ralization	ADE & Anti-S IgG	Female	Male	Total
2019	0.0% (0/28)	0.0% (0/28)	0.0% (0/28)	3.6% (1/28)	0.0% (0/28)	0.0% (0/28)	50.0% (14/28)	50.0% (14/28)	100.0% (28/28)
2020	0.0% (0/24)	0.0% (0/24)	12.5% (3/24)	0.0% (0/24)	0.0% (0/24)	0.0% (0/24)	50.0% (12/24)	50.0% (12/24)	100.0% (24/24)
2021	14.1% (14/99)	4.0% (4/99)	37.4% (37/99)	1.0% (1/99)	3.0% (3/99)	14.1% (14/99)	46.5% (46/99)	53.5% (53/99)	100.0% (99/99)
2022	45.5% (30/66)	42.4% (28/66)	100.0% (66/66)	9.1% (6/66)	30.3% (20/66)	45.5% (30/66)	57.6% (38/66)	42.4% (28/66)	100.0% (66/66)
2023	97.0% (97/100)	98.0% (98/100)	100.0% (100/100)	26.0% (26/100)	95.0% (95/100)	97.0% (97/100)	52.0% (52/100)	48.0% (48/100)	100.0% (100/100)
2024	89.6% (120/134)	90.3% (121/134)	100.0% (134/134)	16.4% (22/134)	88.8% (119/134)	89.6% (120/134)	47.8% (64/134)	52.2% (70/134)	100.0% (134/134)
2025	89.8.0% (44/49)	93.9% (46/49)	100.0% (49/49)	10.2% (5/49)	81.6% (40/49)	91.8% (45/49)	51.0% (25/49)	49.0% (24/49)	100.0% (49/49)
2026	96.7% (29/30)	96.7% (29/30)	100.0% (30/30)	36.7% (11/30)	93.3% (28/30)	96.7% (29/30)	40.0% (12/30)	60.0% (18/30)	100.0% (30/30)

**Figure 11:**
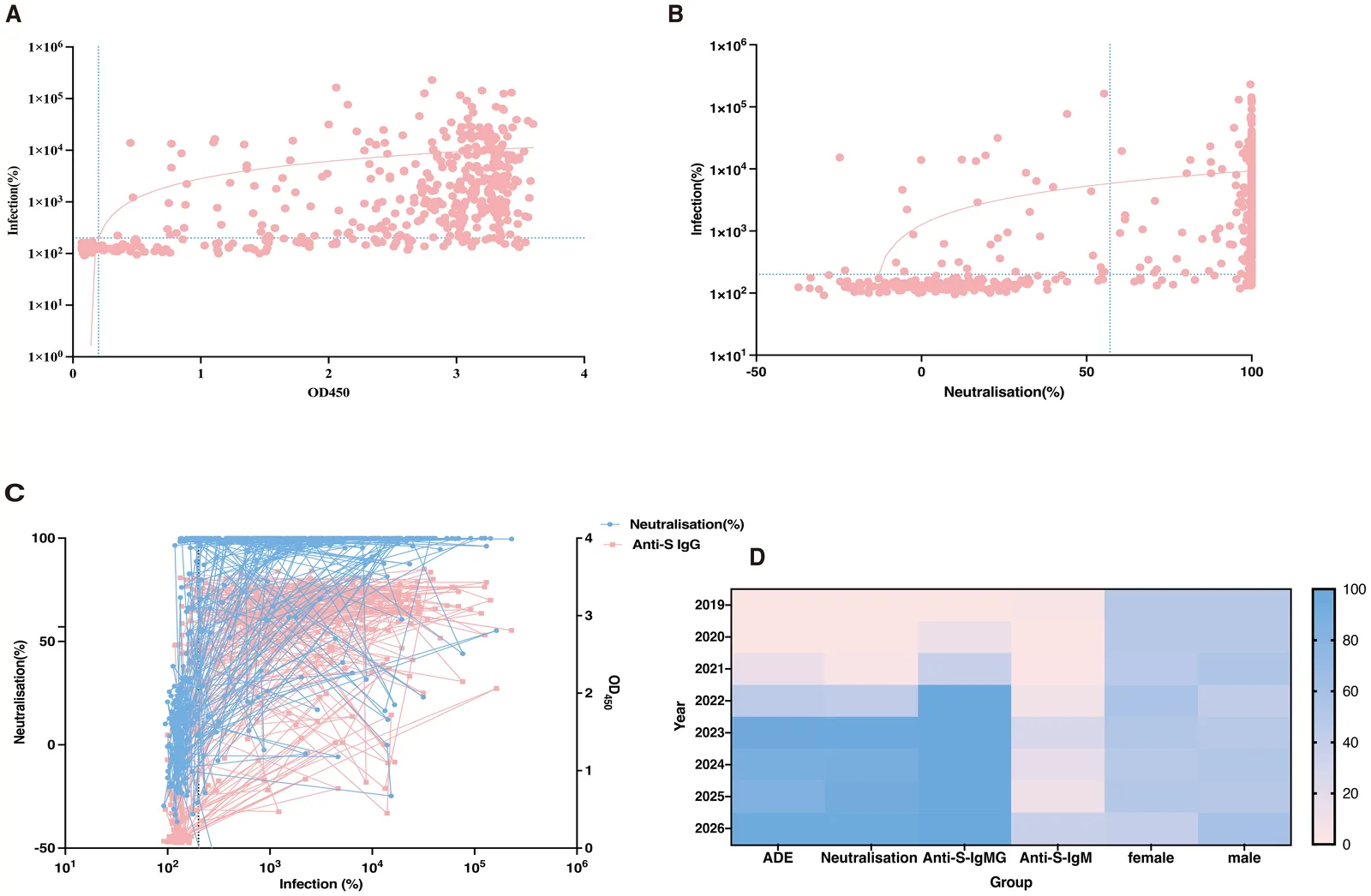
Statistical analysis of ADE results in serum. **(A)** Scatter plot showing the correlation between ADE assay results and Anti-S IgG test results, with Anti-S IgG test results (OD_450_) on the X-axis and ADE infection rate results on the Y-axis; **(B)** Scatter plot showing the correlation between ADE assay results and neutralization assay results, with serum neutralization capacity on the X-axis and ADE results on the Y-axis; **(C)** The X-axis represents ADE infection rate results; the left Y-axis shows neutralization assay results, and the right Y-axis shows Anti-S IgG results. No significant differences were observed between the results. **(D)** A heatmap illustrating the relationship between ADE occurrence and year, serum Anti-S-IgG and Anti-S-IgM antibody levels, and gender. Source data are provided as a source data file.

Finally, we performed correlation analyses using all serum test results (**Figure 11**). We performed point-biserial Pearson correlation analyses to evaluate linear associations between binary ADE status and study variables, with 95% CIs estimated for each correlation coefficient. Anti-S-IgG levels showed a significant positive correlation with ADE (r = 0.660, 95% CI = [0.609, 0.706], *P* < 0.001), suggesting that higher binding antibody levels may contribute to increased Fc-mediated immune complex formation, which in turn could facilitate ADE under subneutralizing conditions. Consistently, sample collection year was also positively correlated with ADE (r = 0.632, 95% CI = [0.578, 0.681], *P* < 0.001), indicating a potential temporal shift in antibody profiles or population immunity background that may influence ADE susceptibility. In contrast, gender showed no significant correlation with ADE (r= -0.048, 95% CI: = [-0.132, 0.380], *P* = 0.272*)*, suggesting that ADE-related responses are largely independent of sex-related immunological differences in this cohort.

To further investigate changes in immune regulation in sera from patients with different degrees of ADE, we selected 175 serum samples from individuals with varying degrees of ADE severity between 2019 and 2026 (**Supplementary Figure S2**) and conducted a direct comparison between the cytokine profiles and the severity of ADE. (**Figure 12**). IL-12p70 exhibited more robust changes (**Figure 12A**). The concentration of IL-12p70 in the Mid group was significantly higher than that in the Control group (*P* < 0.01) and the Model group (*P* < 0.0001); and the IL-12p70 levels in the Low group were also higher than those in the Model group (*P* < 0.05). In sharp contrast, TNF-α levels in the Mid group were significantly lower than those in the Control and Model groups (both *P* < 0.05) (**Figure 12B**). The main data points for TNF-α in the Low, Mid, and High groups were all significantly lower (concentrated between 0 and 0.3), and the degree of dispersion was significantly reduced. For cytokines such as IFN-γ, IL-1β, IL-2, IL-4, IL-6, IL-8, IL-10, and IL-13, the results showed no statistically significant differences among the groups (*P* > 0.05). In summary, the sera that could induce ADE mainly showed changes in IL-12p70 and TNF-α, while no significant effects were observed on other inflammatory-related factors. The differential regulation of IL-12p70 and TNF-α suggests that ADE may modulate the immune microenvironment by selectively regulating specific cytokine networks, rather than through a simple pro-inflammatory or anti-inflammatory effect.

**Figure 12:**
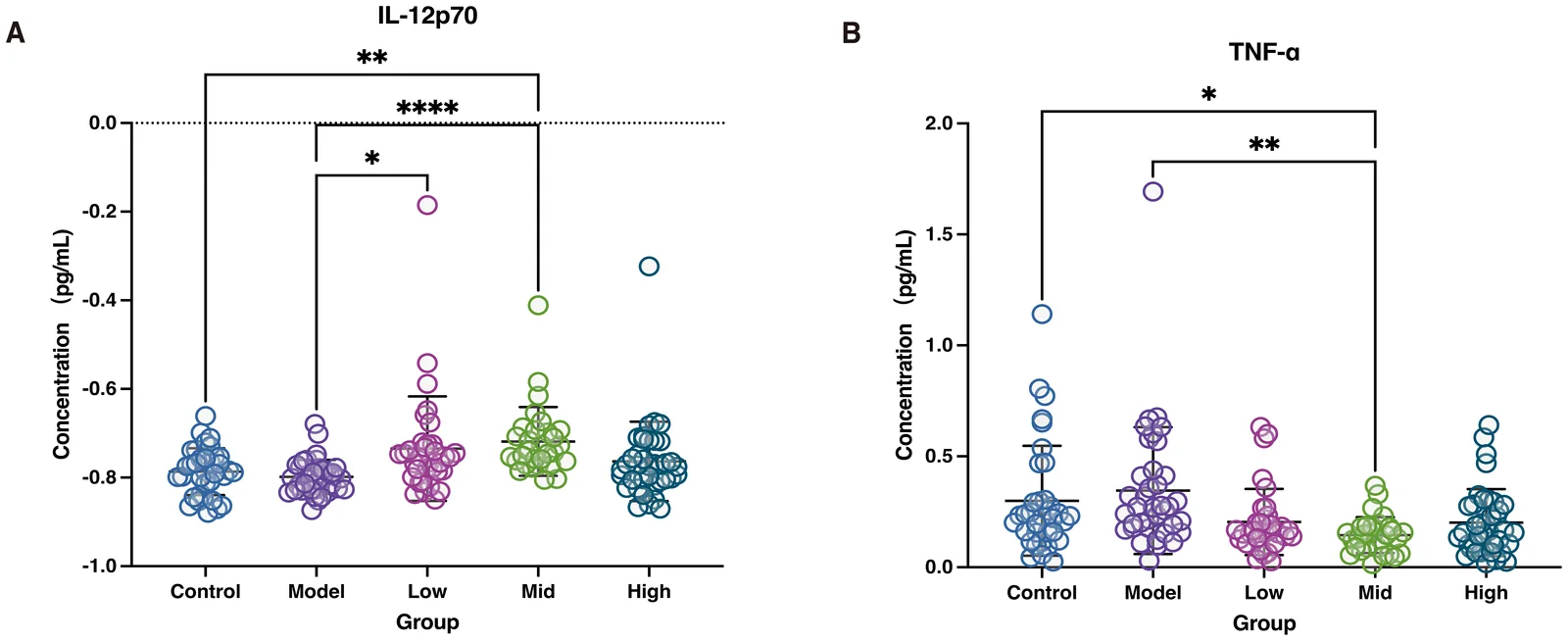
Serum cytokine profiles associated with different degrees of ADE. **(A)** Serum IL-12p70 concentrations measured using a chemiluminescence immunoassay (MSD, Meso Scale Discovery). Significant differences were observed between groups (one-way ANOVA followed by Tukey’s multiple comparisons test): Mid group versus Control group (*p* = 0.0016), Low-ADE group versus Model group (*p* = 0.0432), and Mid-ADE group versus Model group (*P* < 0.0001). **(B)** Serum TNF-α concentrations measured using the same platform. Significant differences were detected between the Mid-ADE group and the Control group (*p* = 0.0114), and between the Mid-ADE group and the Model group (*p* = 0.0017). Participants were stratified into five groups based on anti-S IgG levels and ADE: Control group (Anti-S-IgG OD_450_ < 0.2, no antibody and no ADE, n = 35); Model group (Anti-S-IgG OD_450_> 0.2, antibody-positive without ADE, n = 38); Low-ADE group (Anti-S-IgG OD_450_ > 0.2, infection rate of ADE 200–500%, n = 34); Mid-ADE group (Anti-S-IgG OD_450_ > 0.2, infection rate of ADE 500–2,000%, n = 30); and High-ADE group (Anti-S-IgG OD_450_ > 0.2, infection rate of ADE > 2,000%, n = 38). Data are presented as individual values with mean indicated. Each dot represents one sample. Statistical significance is indicated as *P < 0.05, **P < 0.01, ****P < 0.0001 in Welch’s one-way ANOVA with Games–Howell *post-hoc* multiple comparisons. Dots and error bars indicate the means and standard deviations of triplicate wells, respectively. Data represent the mean ± SD. Source data are provided as a source data file.

## Discussion

ADE refers to the phenomenon in which subneutralizing and non-neutralizing antibodies mediate viral entry into immune cells via FcγR, thereby enhancing viral replication and exacerbating disease progression (21). This mechanism was first elucidated in DENV: immune complexes formed by heteroserotype antibodies can promote viral internalization and induce severe disease (22). *In vitro* studies have further demonstrated that ADE primarily relies on the FcγR pathway rather than classical receptor-mediated entry (23). Therefore, elucidating the balance between the dual effects of antibodies—protection and pathogenesis—constitutes the core theoretical foundation for revealing the mechanisms of ADE in respiratory coronaviruses.

Previous studies have demonstrated that activating FcγR is the key receptor mediating ADE; for example, in a DENV model, immune complexes promote viral internalization via FcγRIIA/III (24, 25). FcγRIIA and FcγRIIIA have been identified as the primary receptors mediating SARS-CoV-2 ADE (26, 27). Building on these findings, this study further confirms that the inhibitory receptor FcγRIIB can also mediate ADE for both SARS-CoV-2 and MERS-CoV, and for the first time includes it in the receptor repertoire of MERS-CoV. Notably, within our overexpression cell-based system, notably, FcγRIIB mediates ADE over a broader range of effective antibody concentrations and exhibits a stronger enhancing effect than FcγRIIA, suggesting that FcγRIIB may potentially support ADE across a broader range of target cell lineages and thereby expand the receptor framework implicated in ADE by respiratory coronaviruses.

The mechanism underlying ADE is not solely determined by receptor selectivity. Previous epigenomic studies have confirmed that the structural characteristics of antibodies and the epitopes they recognize also play a key role in this process (15). Our results further indicate that antibodies targeting specific regions of the viral spike protein (particularly the receptor-binding domain) can significantly induce ADE against SARS-CoV-2 and MERS-CoV in CHO cells expressing high levels of FcγR. This suggests that the occurrence of ADE depends not only on the binding of antibodies to FcγR but is also determined by the epitopes recognized by the antibodies. The conformation of the immune complex formed by RBD-specific antibodies may regulate interactions with FcγR in different ways, thereby determining the initiation and severity of ADE.

The occurrence of ADE follows a typical nonlinear “bell-shaped” curve, primarily occurring in the range of subneutralizing antibody concentrations (15, 25, 28, 29). This study not only validated this pattern but also revealed that it is viral load-dependent: both the antibody concentration that induces the strongest ADE and the peak ADE level increase in tandem with rising viral load. This suggests that ADE is not determined by a single antibody threshold but rather results from a dynamic equilibrium between the virus and antibodies, with its risk window evolving in tandem with viral replication and the host immune response.

Viral entry typically involves steps such as endocytosis, endosome maturation, and lysosomal fusion; this mechanism also applies to FcγR-mediated ADE (30–33). Previous studies have shown that during FcγR-mediated ADE by SARS-CoV-2, the virus-antibody complex, after entering the cell via endocytosis, is transported to lysosomes where it undergoes membrane fusion and viral genome release (34). Building on these findings, to elucidate the intracellular mechanisms of ADE in respiratory coronaviruses, we conducted a systematic analysis using inhibitors targeting NRP1, endosomal acidification, Furin, Cathepsin, and PIKfyve. The results show that although the virus-antibody complex enters cells via FcγR, subsequent infection remains strictly dependent on the classical endosomal transport and proteolytic processing pathways. This suggests that the intracellular mechanisms underlying ADE in SARS-CoV-2 and MERS-CoV overlap significantly with those of classical receptor-mediated infection; the difference between the two lies solely in the initial binding receptor (FcγR vs. ACE2/DPP4), rather than in downstream intracellular processes.

Previous studies have shown that antibodies in the serum of convalescents and vaccinated individuals can mediate SARS-CoV-2 infection of immune cells and induce abnormal immune responses, suggesting that ADE is closely associated with serum antibody characteristics (35–37). Concurrently, with repeated exposure through natural infection and vaccination, changes in the antibody profile and affinity maturation may lead to the emergence or amplification of ADE signals (36), indicating that the dynamic evolution of antibodies is a key factor influencing ADE. Based on this, the present study conducted a retrospective analysis of 530 individual serum samples collected between 2019 and 2026, extending the ADE research from *in vitro* mechanisms to population-based validation. Cross-analysis of ELISA and cell-based assays revealed that the incidence of ADE was highly correlated with the prevalence of anti-S protein antibodies, showing an upward trend over time before eventually leveling off. This finding is consistent with the elevation in antibody levels observed in global populations due to repeated exposure (38–42).It is worth noting that gender did not significantly influence the occurrence of ADE (*P* > 0.05) (42), suggesting that ADE is primarily determined by the quality of the antibody response, its dynamic evolution, and viral characteristics, rather than by the host’s gender (43).

Given the complexity of serum components and the dual nature of the ADE mechanism (exogenous infection and endogenous immune regulation) (44), we performed cytokine analysis on 175 serum samples from the 2019–2026 cohort (102 ADE-positive and 73 ADE-negative cases). The results of IL-12p70 levels showed an increasing trend across serum samples of inducing different degrees of ADE. This pattern may reflect changes in Th1-associated and pro-inflammatory immune responses accompanying ADE, although the underlying biological significance and causal relationship remain to be determined. As a key driver of Th1 differentiation, elevated IL-12p70 levels may reflect the body’s attempt to clear the virus but may also lead to immunopathological damage (45). In contrast, there were no significant changes in IL-4 and IL-10 levels, suggesting that this imbalance does not stem from a Th1/Th2 shift but is more likely due to abnormal pro-inflammatory/anti-inflammatory regulation. It is worth noting that previous studies have suggested that elevated TNF-α levels may have a protective effect during the progression of inflammation (46). Combined with the findings from this study—which showed significantly reduced TNF-α levels and a negative predictive association with the occurrence of ADE—this suggests that elevated TNF-α levels may be one of the potential factors associated with a reduced risk of ADE; however, the specific regulatory mechanisms of TNF-α in ADE require further investigation.

In summary, this study provides a systematic analysis of ADE in respiratory coronaviruses from multiple perspectives, including receptor selectivity, epitope characteristics, and intracellular mechanisms. We not only confirmed that FcγRIIB is a key receptor mediating ADE for SARS-CoV-2 and MERS-CoV—marking its first inclusion in the MERS-CoV receptor family—but also found that it outperforms FcγRIIA in terms of effective antibody concentration range and potentiation effects, thereby expanding the framework of ADE receptors. Mechanistically, ADE relies on immune complexes formed between RBD-specific antibodies and the virus and is regulated by a dynamic equilibrium between viral load and antibody levels, resulting in a nonlinear risk window. A retrospective analysis of 530 serum samples further confirmed that ADE is closely associated with antibody dynamics and is accompanied by immune regulation characterized by an imbalance between IL-12p70 and TNF-α. As the antibody profile evolves due to natural infection and vaccination, the widespread presence of subneutralizing antibodies may increase the potential risk of ADE (12, 13, 47, 48). However, establishing the translational relationship between *in vitro* ADE and clinically relevant disease remains a significant challenge. Our single-cycle pseudovirus system primarily assesses antibody-dependent enhancement of viral entry; increased luciferase activity indicates enhanced viral entry but does not reflect productive viral replication, progeny virus release, or downstream cytokine responses. In addition, the archived serum samples from 2019, 2020, 2025 and 2026 have limited stock and cannot be replenished retrospectively, leading to unbalanced sample sizes across years, which should be interpreted with caution. Therefore, confirmation of clinically relevant ADE will require complementary studies using replication-competent viruses and appropriate primary target cells. Further research is also needed to clarify FcγRIIB-mediated signaling and the ADE characteristics of different viral variants. Notably, our findings suggest that naturally acquired antibodies in humans may still retain the capacity to mediate ADE *in vitro* within the evolving epidemiological landscape up to 2026. These findings highlight the importance of distinguishing enhanced viral entry from productive infection and immunopathology and may inform the rational design of epitopes and immunization strategies to maximize protective immunity while minimizing potential risks.

## Conclusions

This study establishes a mechanistic and epidemiological framework for coronavirus ADE, demonstrating that epitope-specific antibodies can promote FcγR-dependent viral entry and inflammatory activation. These findings provide important insights for evaluating vaccine safety, optimizing antibody-based therapies, and understanding antibody-mediated immune regulation during coronavirus infections.

## Data Availability

The original contributions presented in the study are included in the article/**Supplementary Material**. Further inquiries can be directed to the corresponding author/s.

## Supplementary material

Supplementary Figure 1 ADE profiles of 175 human serum samples across serial dilutions. Human serum samples (n = 175) were serially diluted (1:20–1:20480) and evaluated for antibody-dependent enhancement (ADE) activity. Samples were classified into five groups based on their ADE phenotypes: **(A)** Control (n = 35), **(B)** Model (n = 38), **(C)**. Low ADE (n = 34), **(D)**. Mid ADE (n = 30), and **(E)**. High ADE (n = 38). Infection percentages at each serum dilution are presented for individual samples, demonstrating distinct ADE response patterns and dilution-dependent enhancement effects among groups. Dots and error bars indicate the means and standard deviations of triplicate wells, respectively. Data represent the mean ± SD. Source data are provided as a source data file.
