# Supplementary material for: Integrated mechanistic and serological analyses reveal determinants of antibody-dependent enhancement in respiratory coronaviruses
Source: Front Immunol. 2026 Oct 23;17:1967945. doi: 10.3389/fimmu.2026.1967945 (PMC13739713; doi:10.3389/fimmu.2026.1967945)
Supplement: Supplementary file 1 [file DataSheet1.pdf]

Supplementary Information

Appendix 1

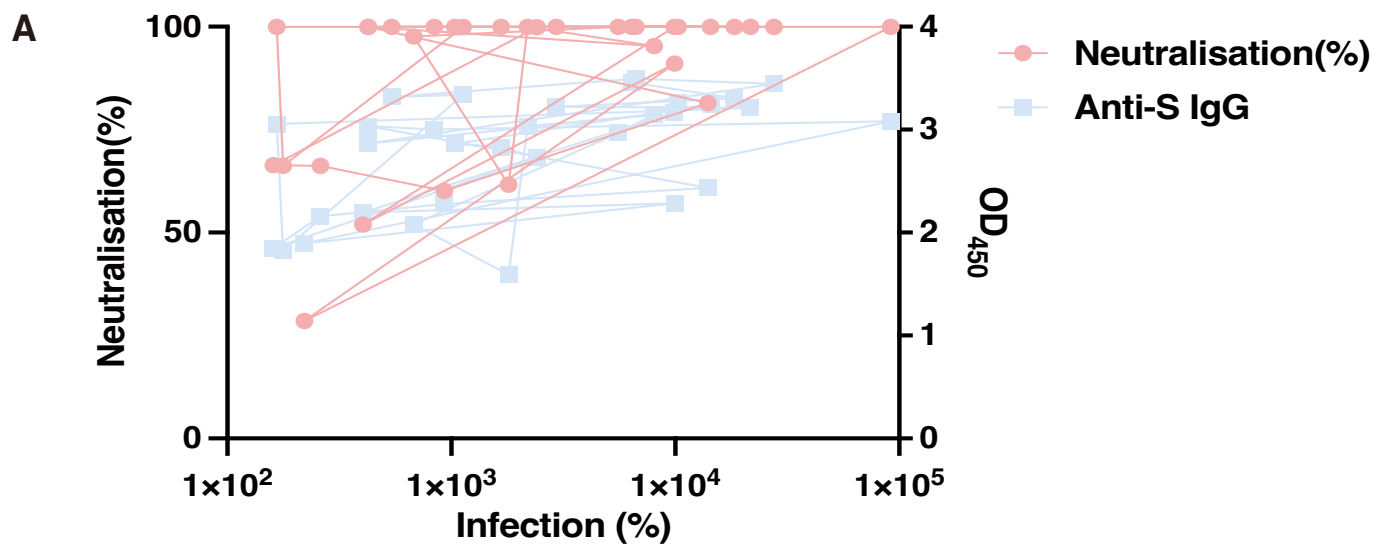

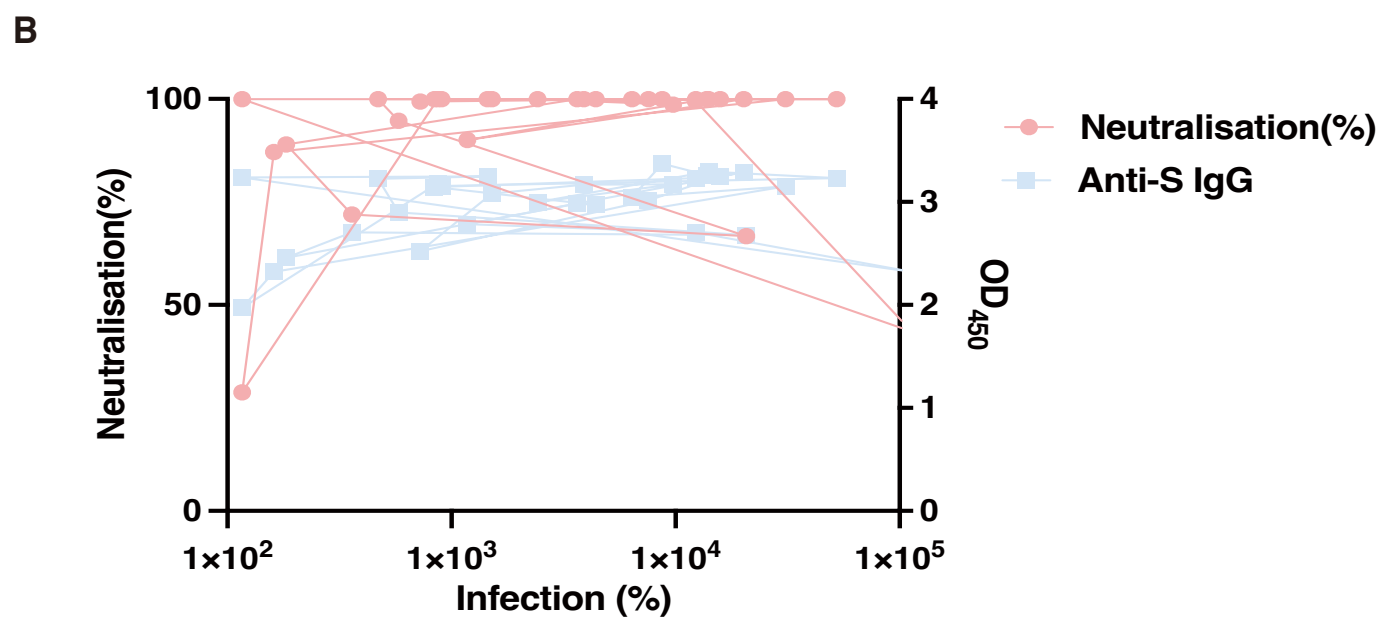

Supplementary Figure 1. Reproducibility of ADE, anti-S IgG, and neutralization assays across two testing rounds in 55 serum samples. A. Results of the first round of ADE, anti-S-IgG, and neutralization assays for the 55 sera collected in 2024; **B.** Results of the second round of ADE, anti-S-IgG, and neutralization assays for the 55 sera collected in 2024. Dots and error bars indicate the means and standard deviations of triplicate wells, respectively. Data represent the mean ± SD. Source data are provided as a source data file.

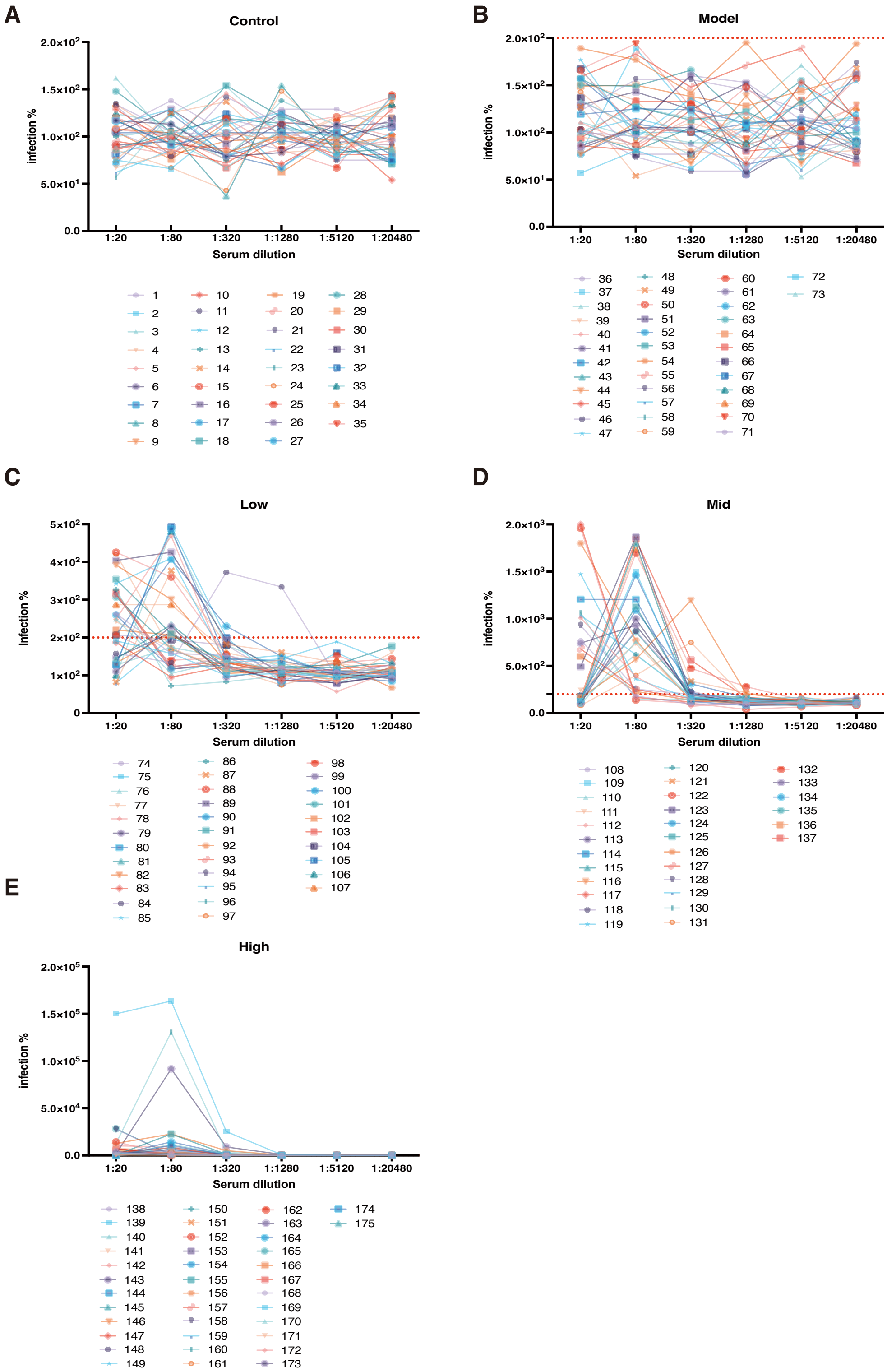

Supplementary Figure 2. ADE profiles of 175 human serum samples across serial dilutions. Human serum samples (n = 175) were serially diluted (1:20–1:20480) and evaluated for antibody-dependent enhancement (ADE) activity. Samples were classified into five groups based on their ADE phenotypes: **A.** Control (n = 35), **B.** Model (n

= 38), **C.** Low ADE (n = 34), **D.** Mid ADE (n = 30), and **E.** High ADE (n = 38). Infection percentages at each serum dilution are presented for individual samples, demonstrating distinct ADE response patterns and dilution-dependent enhancement effects among groups. Dots and error bars indicate the means and standard deviations of triplicate wells, respectively. Data represent the mean ± SD. Source data are provided as a source data file.
